# Proteomic analysis of Medulloblastoma reveals functional biology with translational potential

**DOI:** 10.1186/s40478-018-0548-7

**Published:** 2018-06-07

**Authors:** Samuel Rivero-Hinojosa, Ling San Lau, Mojca Stampar, Jerome Staal, Huizhen Zhang, Heather Gordish-Dressman, Paul A. Northcott, Stefan M. Pfister, Michael D. Taylor, Kristy J. Brown, Brian R. Rood

**Affiliations:** 1 Center for Cancer and Immunology Research, Children’s Research Institute, Children’s National Health System, Washington, DC, USA; 2 Center for Genetic Medicine, Children’s Research Institute, Children’s National Health System, Washington, DC, USA; 3 Department of Pediatric Hematology and Oncology, Hopp Children’s Cancer Center at the NCT Heidelberg (KiTZ), Division of Pediatric Neurooncology, German Cancer Research Center (DKFZ), Heidelberg University Hospital, Heidelberg, Germany; 4 Developmental and Stem Cell Biology Program, Hospital for Sick Children, Toronto, ON Canada; 5 The George Washington University School of Medicine and Health Sciences, Washington DC, USA

**Keywords:** SILAC, Proteomics, Medulloblastoma, Pediatric brain tumor

## Abstract

Genomic characterization has begun to redefine diagnostic classifications of cancers. However, it remains a challenge to infer disease phenotypes from genomic alterations alone. To help realize the promise of genomics, we have performed a quantitative proteomics investigation using Stable Isotope Labeling by Amino Acids in Cell Culture (SILAC) and 41 tissue samples spanning the 4 genomically based subgroups of medulloblastoma and control cerebellum. We have identified and quantitated thousands of proteins across these groups and find that we are able to recapitulate the genomic subgroups based upon subgroup restricted and differentially abundant proteins while also identifying subgroup specific protein isoforms. Integrating our proteomic measurements with genomic data, we calculate a poor correlation between mRNA and protein abundance. Using EPIC 850 k methylation array data on the same tissues, we also investigate the influence of copy number alterations and DNA methylation on the proteome in an attempt to characterize the impact of these genetic features on the proteome. Reciprocally, we are able to use the proteome to identify which genomic alterations result in altered protein abundance and thus are most likely to impact biology. Finally, we are able to assemble protein-based pathways yielding potential avenues for clinical intervention. From these, we validate the EIF4F cap-dependent translation pathway as a novel druggable pathway in medulloblastoma. Thus, quantitative proteomics complements genomic platforms to yield a more complete understanding of functional tumor biology and identify novel therapeutic targets for medulloblastoma.

**Electronic supplementary material:**

The online version of this article (10.1186/s40478-018-0548-7) contains supplementary material, which is available to authorized users.

## Introduction

In a series of landmark papers, microarray transcriptome characterization subdivided the malignant childhood brain tumor medulloblastoma into at least four distinct entities [11, 31, 32, 49, 66]. Chromosomal copy number alterations, clinical characteristics such as age and survival, and methylation array data correlated well with the transcriptomic classification, reinforcing the identity of distinct subgroups of the disease [50]. However, the real goal of more informative, biologically based tumor taxonomy is to drive discovery of new therapeutic interventions. The WNT and SHH pathways drive their eponymous subgroups and their activity is enhanced by mutations in key elements of the pathways. But the other two subgroups, 3 and 4, do not harbor mutations at the same frequency and present a greater challenge when trying to discern their biological determinants from gene expression signatures alone [48]. Largely, this difficulty emanates from the central assumption of transcriptomics, that a gene’s expression level predicts its contribution to cell biology. We have therefore applied quantitative proteomics in an attempt to replace this assumption with knowledge about the proteome, as proteins more directly determine the functional state of the cell.

The work of the cell is performed largely by proteins. While protein production begins with gene transcripts, a growing literature has demonstrated that transcript abundance does not predict protein quantity for a large majority of genes [21, 24, 77]. A large number of post-transcriptional regulatory mechanisms from interfering RNAs to selective RNA binding proteins affect differential translation in response to a wide range of cellular conditions [63]. The first paper published from the CPTAC (Clinical Proteomic Tumor Analysis Consortium) initiative showed a correlation coefficient of 0.23 between transcript and protein abundance [77] and subsequent publications have replicated this experience [45, 78]. It is also becoming increasingly recognized that cancer cells utilize alternative pathways of translation initiation that prioritize proteins important in the cellular response to stress [41, 60, 63]. Thus, a true picture of the cancer cells’ functional state must be based not only upon genomic features but also include the protein complement if we are to decipher its biology. Further, and most importantly, in order to interfere with that biology, proteins remain the most actionable targets of pharmaceutical and immunotherapeutic intervention.

While proteomics can point the way forward toward translational progress, it can also provide a valuable lens through which to view cancer genomics. Genomic technology generates rich datasets with little inherent means to determine the relative importance of each individual finding. In contrast, clinical proteomics data is much less complete but is, by its nature, more representative of cellular biology. By employing the axiom that for a genomic event to influence phenotype it must be translated into the proteome, one can apply a filter to help discern signal from noise in the massive datasets produced by genomics platforms. In this way, these two areas of study can complement one another. Here, we present the first proteogenomic characterization of pediatric medulloblastoma and demonstrate the unique contributions to biologic understanding offered by quantitative proteomics.

## Results

### Quantitative proteomics of medulloblastoma

**Fig. 1:**
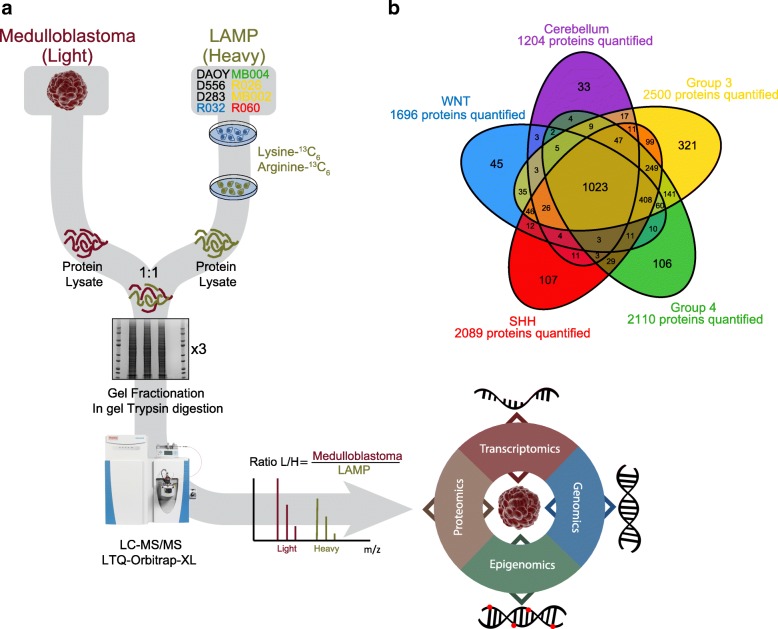
SILAC proteomics workflow and output. **a** The Labeled Atlas of Medulloblastoma Proteins (LAMP) was generated by combining equal amounts of isotopically labeled (Lysine-^13^C_6,_ and Ariginine^13^C_6_) proteins from 8 primary and established cell lines, representing the four primary medulloblastoma subgroups. Protein lysates from tissues were spiked 1:1 with the SILAC reference atlas (LAMP). The resulting protein lysates were fractionated on a 1-D gel in triplicate, trypsin digested, and further fractionated by HPLC in line with the MS/MS analysis. The spectra were then searched against the Uniprot or custom protein databases to identify peptide sequences and their originating proteins. Protein quantitation is derived from the ratio of the light tissue peptides relative to the heavy SILAC reference peptides. The proteomic data was functionally integrated with tumor-matched transcriptomic, epigenomic, and genomic data. **b** Venn diagram of quantified proteins along the 4 medulloblastoma subgroups and cerebellum tissues. A total of 2901 proteins were quantified

The ability to simultaneously identify and quantitate proteins in a given tissue is critical to an attempt to characterize the proteome of a tumor. For this purpose, we applied Stable Isotope Labeling by Amino acids in Cell culture (SILAC) [12, 20] to clinical samples of 36 medulloblastoma tumors and 5 control samples of normal cerebellum (Additional file 1: Table S1). For our studies, we created a pooled super-SILAC reference atlas, termed the Labeled Atlas of Medulloblastoma Proteins (LAMP), containing labeled proteins from 8 primary and established cell lines chosen to represent the breadth of medulloblastoma across the four genomic subgroups (Methods). The LAMP was then spiked at a ratio of 1:1 into tumor tissue protein lysates and liquid chromatography-tandem mass spectrometry (LC-MS/MS) was performed (Fig. 1a and Methods). We identified a total of 54,403 unique peptides among the 41 samples, corresponding to 4,987,397 spectra in an assembly of 2787 protein groups with a 5% protein false discovery rate (Methods). To facilitate integration with genomic data, we assigned the same quantification value to each protein belonging to the same protein group. In this way, we were able to relatively quantitate 2901 proteins across the 41 samples. 1023 (37%) of the proteins were common to both medulloblastoma and control cerebellum while 408 (15%) proteins were shared by all medulloblastoma subgroups. Group 3 exhibited the most unique proteins (11.5%) and groups 3 and 4 had the highest number of shared proteins of any 2 subgroups (Fig. 1b). These data indicate that the LAMP was capable of yielding quantitative protein data across the four genomic subgroups of medulloblastoma.

### Correlation between mRNA and protein abundance.

**Fig. 2:**
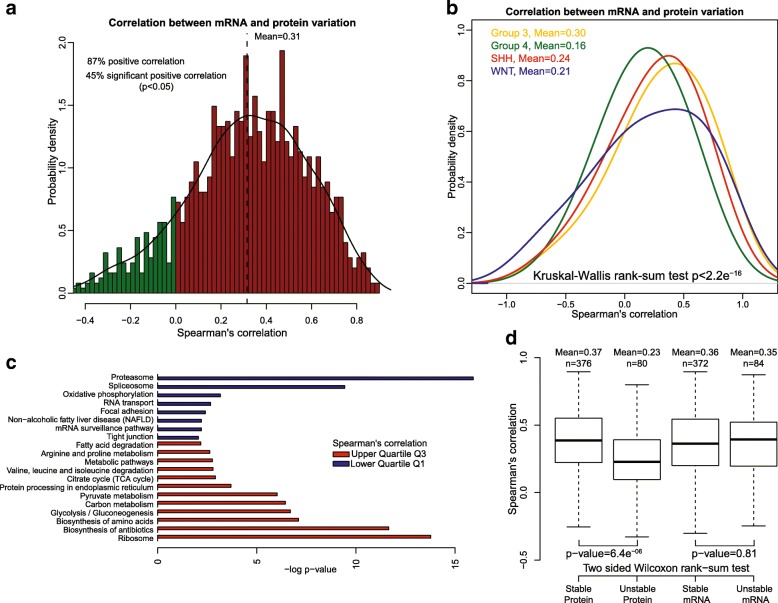
Correlation between mRNA and protein abundance. **a** mRNA and protein were positively correlated for most (87%) pairs of mRNA-proteins with a mean Spearman’s correlation of 0.31, but only 45% showed a significant correlation (*p* < 0.05). **b** Density plot of Spearman’s correlation by medulloblastoma subgroup; a significantly lower Spearman correlation mean was found in Group 4, 0.16 compared to 0.30, 0.24 and 0.21 in Group3, SHH and WNT respectively. **c** Different biological processes displayed differences in mRNA and protein correlation. Genes encoding ribosomal and metabolic functions showed higher mRNA-protein correlation than those involved in the spliceosome or proteasome. **d** Relationship between mRNA-protein correlation and the stability of the molecules. Human mRNA and proteins are divided into stable or unstable and the distribution of mRNA-protein Spearman’s correlation is represented by box-plots. Unstable proteins have significantly lower correlations than stable ones; however, no differences were found for mRNA. *p*-values were calculated using the two-sided Wilcoxon rank sum test

To better understand the extent to which transcript level predicts protein abundance, we calculated the concordance between mRNA and protein abundance for the 1240 individual genes that had suitable mRNA and protein measurements in 35 matched tumor samples (Methods and Additional file 2: Table S2). Although 87% of the genes had a positive mRNA-protein correlation, only 45% had statistically significant correlations (*p*-value < 0.05) (Fig. 2a and Additional file 2: Table S2). The average Spearman’s correlation between mRNA and protein variation was 0.31, comparable to that reported previously in colorectal, breast and ovarian cancer [45, 77, 78]. A similar analysis for each medulloblastoma subgroup showed that group 4 tumors demonstrate the lowest concordance (Fig. 2b and Additional file 3: Figure S1b), with a Spearman correlation mean of 0.16 compared to 0.30, 0.24, and 0.21 for group 3, SHH, and WNT respectively (four group comparison, Kruskal-Wallis test, *p*-value < 2.2e^− 16^).

To test whether mRNA-protein correlation is related to the biological function of the protein, we performed KEGG Pathways enrichment analysis. We found that gene/protein pairs with higher correlation (greater than the Upper Quartile Q3) were enriched in metabolic and ribosomal pathways, whereas there was a low correlation for genes/proteins enriched in proteasome, spliceosome, oxidative phosphorylation, and RNA transport pathways (Fig. 2c).

Among other factors such as secondary mRNA structure, codon bias, ribosomal density, regulatory proteins and RNAs [40], the half-life of proteins and mRNA is a major factor influencing mRNA-protein correlation. From studies on mammalian cells, it is reported that proteins are 5 times more stable and 900 times more abundant than mRNA while spanning a lower dynamic range [58]. To analyze the effect of mRNA and protein half-lives on mRNA-protein correlation, we used half-life data generated from mammalian cells by parallel metabolic pulse labeling [58]. We found that genes with stable protein products tend to have higher mRNA-protein correlation than those with unstable proteins, however we found no differences between stable and unstable mRNA (*p*-values 6.4e^− 06^ and 0.81 respectively, two-sided Wilcoxon rank sum test) (Fig. 2d). This finding is similar to colon cancer in which the protein-mRNA correlation was found to be highest when both mRNAs and proteins were stable [77]. These results demonstrate that mRNA transcript abundance is a poor predictor of protein complement and that both the biological function of proteins and their rate of turnover likely impact mRNA-protein correlation. Thus, extreme caution should be exercised when predicting biologic phenotype from gene expression data alone.

### Effect of methylation and copy number on protein expression.

To study the effect of DNA methylation on protein expression, we calculated the differentially methylated regions (DMR) by comparing each medulloblastoma subgroup to control cerebellum samples. Differentially expressed proteins and genes were calculated in the same way. We found that the number of differentially expressed genes (mRNA) associated with a DMR was higher than the number of differentially expressed proteins associated with a DMR across all subgroups (Additional file 4: Figure S2a). We then analyzed genes with an associated DMR to correlate loss/gain of methylation with mRNA or protein expression/repression respectively. We found that for all subgroups both mRNA and protein expression showed odds ratios greater than 1, however only the mRNA correlations were significant (*p*-value < 0.01, Fisher exact test) (Additional file 4: Figure S2b). Together, these results suggest that there is a poor correlation between epigenetic programs, evidenced by DNA methylation, and protein quantity, indicating that additional regulation at the level of translation and/or protein stability governs protein abundance.

**Fig. 3:**
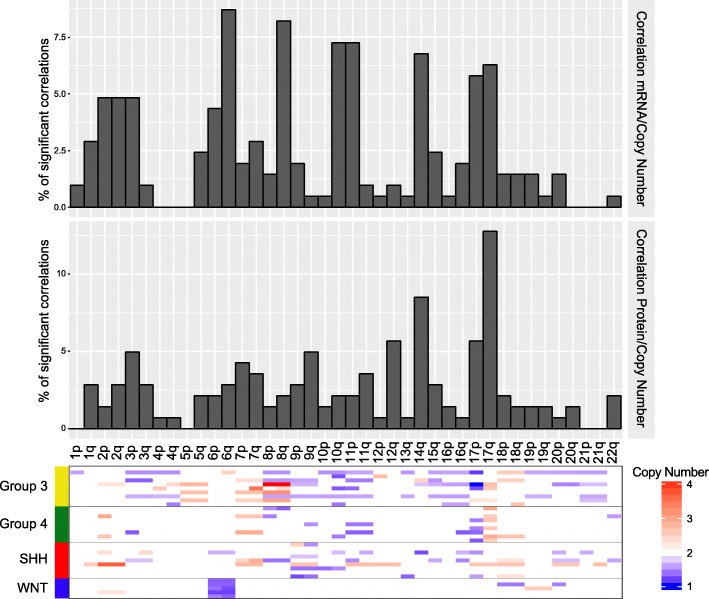
Effect of copy number alteration on MRNA and protein abundance. Frequency of significant correlations between CNA and mRNA (upper panel) or protein (middle panel) across all chromosomes. The heatmap (lower panel) indicates the copy number for each sample

To analyze the impact of copy number alterations (CNA) on protein and mRNA expression, we calculated the correlation between copy number alterations, mRNA and protein quantity for 1147 genes. Probability density showed that although 81 and 69% of the genes showed positive CNA-mRNA and CNA-protein correlation respectively, only 18 and 13% had statistically significant Spearman’s correlations (*p*-value < 0.05). Among the genes with significantly correlated CNA-mRNA and CNA-protein, 98 and 90% showed a positive correlation (Additional file 5: Figure S3a and Additional file 6: Table S3). These results show greater correlation between copy number and mRNA than CNA-protein as has also been shown in colon and rectal cancer [77]. Representation of significantly correlated genes across the chromosomes revealed differences between CNA-mRNA and CNA-protein correlation. While chromosome bands 6q, 8q, 10q, 11p, 14q, 17p and 17q present the highest frequency of significant CNA-mRNA correlations, only chromosome bands 17q and 14q were found to correlate at the protein level (Fig. 3 and Additional file 5: Figure S3b). Some of these chromosome bands correspond to well-known chromosome alterations in medulloblastoma tumors, such as monosomy 6 in WNT subgroup tumors, deletions of 10q and 11p in group 3 tumors, and isochromy of chromosome 17 in group 4 tumors [50]. The gain of chromosome 17q, a frequent event in group 3 and 4 tumors that has been shown to be prognostic [61], was associated with the largest number of significant copy number/mRNA/protein correlations. Among the 56 genes in the 17q region with quantifiable protein measurements, 18 (32%) showed significant CNA-protein alterations, while only 10 (18%) showed significant CNA-mRNA correlation (*p* < 0.05, Spearman’s correlation, Additional file 6: TableS3).

Significant CNA-protein correlations identify amplified sequences that translate to high protein abundance illustrating that proteomic measurements can help to prioritize genes in amplified regions for further examination [77]. Among the 18 genes with significant CNA-protein correlation in chromosome arm 17q, we found two genes that have been previously associated with medulloblastoma biology, LASP1 and GRB2 (Additional file 7: Figure S4a) [27, 67]. Other interesting candidates included KPNB1 and ARHGDIA, both correlating between CNA and protein but not with mRNA level (Additional file 7: Figure S4a). KPNB1 is a nuclear transport receptor, which regulates glioma proliferation via the Wnt/β-Catenin pathway [39]. ARHGDIA, encoding a Rho GDP-dissociation inhibitor, is implicated in cell migration and is upregulated in several cancers including glioma [18]. Interestingly, the ARHGDIA protein is elevated in group 4 tumors where isochrome 17q is frequent (Additional file 7: Figure S4b). In summary, we found more correlations between CNA and mRNA compared with protein levels indicating that in many cases, CNA-driven mRNA transcript alterations do not translate to the abundance of their corresponding protein [77]. Additionally, we have found a high frequency of positive correlations between copy number and protein expression in chromosome arm 17q genes, reinforcing this alteration as an important factor in medulloblastoma biology. When chromosomal alterations involve many genes, proteomics can be instrumental in discerning which genes are most likely to project that alteration’s impact onto cellular biology.

### Proteomic subgroup classification recapitulates genomic subgroups using different data elements

**Fig. 4:**
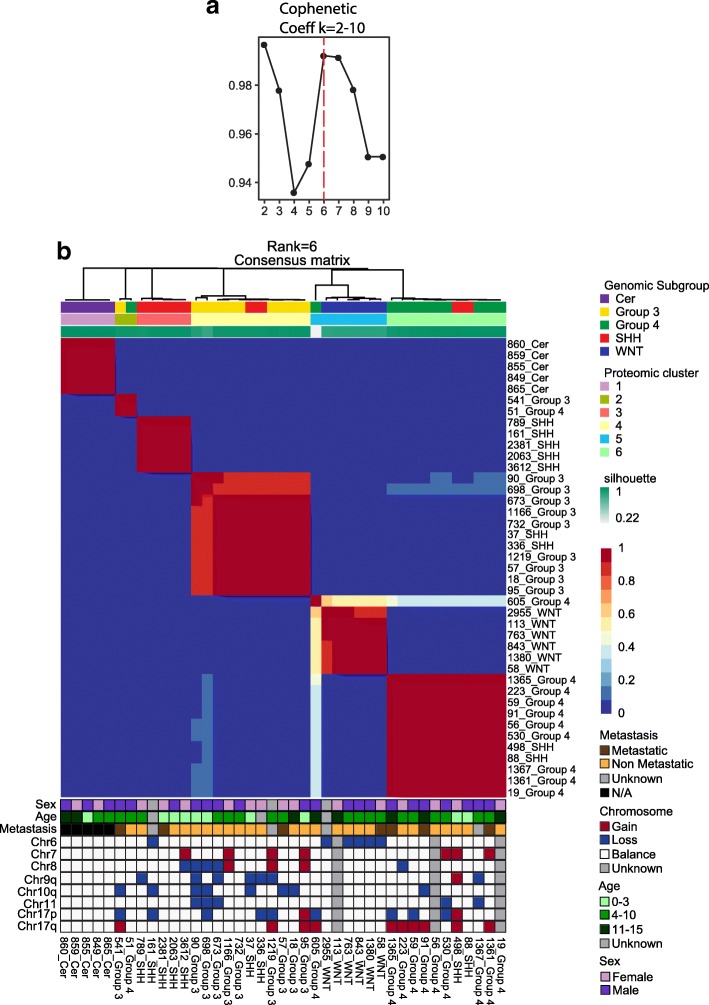
Proteomic subgroup classification recapitulates genomic subgroups. Non-negative matrix factorization consensus clustering of protein expression data from 36 primary medulloblastoma and five normal cerebellar tissues reveals six (Cophenetic Coefficient, k = 6) subgroups. This result largely recapitulates the subgroups found by genomic data

Genomic subgrouping of medulloblastoma has led to many new insights into the cells of origin, active pathways, and varying outcomes to traditional therapies. However, it has proven difficult to translate these findings into new subgroup specific therapies, something for which proteomics may be better suited. To identify molecular subtypes of medulloblastoma using proteomic data, we utilized an unsupervised clustering algorithm based on non-negative matrix factorization (NMF) [16]. When applied to our proteomic expression data consisting of 36 primary medulloblastoma tumors and five healthy cerebellar tissues, the optimal number of clusters in our dataset was determined to be 6 (Fig. 4a). These represent five medulloblastoma subgroups plus one of cerebellum, which approximate the genomic subgroup assignments by nanostring and methylation array based methods [28, 29, 57] (Fig. 4b). The fifth medulloblastoma group contains only 2 tumors, genomically denoted as group 3 and 4. Annotation with DNA copy-number alterations showed that the proteomic subgroups also correlate to high frequency copy number alterations in the same way that the genomic subgroups do [50]. For example, WNT tumors harbor monosomy 6, isolated 17p losses are confined to group3 and i17q is found only in group 4 tumors. While recent analyses of genomic and epigenomic data utilizing large numbers of tumor samples have further subdivided the four main genomic subgroups into 12 subtypes, proteomic analysis with this limited number of samples continues to support the four main genomic subgroups [7].

While the ability to recapitulate the genomic subgroups from proteomic data supports the validity of the subgroups, it is important to note that the proteome classifies tumors based upon different data points. To test this, we attempted to cluster the tumors using the transcript abundance of each of the genes coding for the proteins used in the proteomic classification. That dataset was unable to recreate the subgroups indicating that the proteomic classification draws from a different dataset (Additional file 8: Figure S5). In general, the genes that differentiate between the subgroups have not yielded therapeutic targets, limiting the translational impact of genomic subgrouping. Therefore, the ability of proteomics to differentiate the subgroups using different data points provides an additional translational opportunity.

### Identification of subgroup enriched protein isoforms from unique peptides

**Fig. 5:**
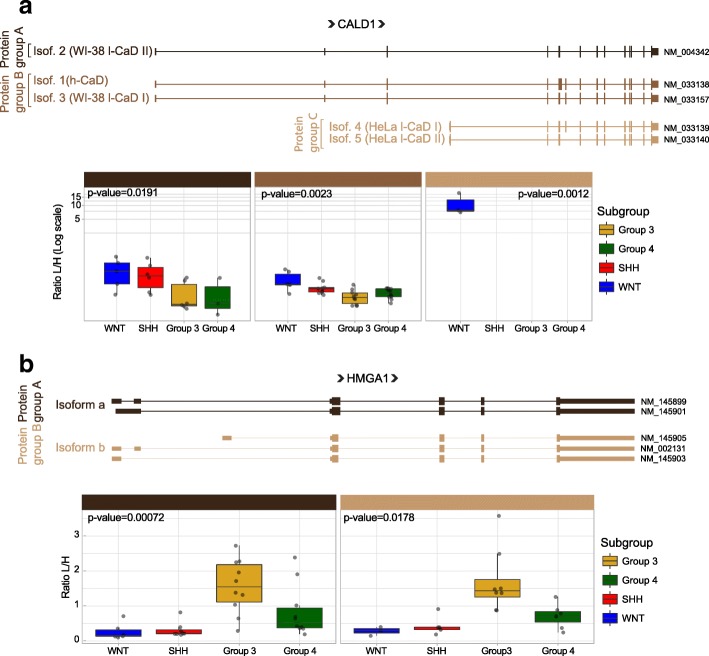
CALD1 and HMGA1 isoforms in medulloblastoma tumors. Schematic representation of CALD1 (**a**) and HMGA1 (**b**) isoforms. The boxplots show the quantification of each protein isoform group across all medulloblastoma subgroups. *p*-values for differences between subgroups were calculated based on the Kruskal-Wallis rank-sum test. A protein group is defined as the group of isoforms that are indistinguishable due to the position of identified peptides

The manually annotated Swiss-Prot Uniprot database does not contain gene isoforms and thus one peptide can map to multiple proteins. Although this database is effective for establishing the general proteome of tumors, it is not sufficient to quantify differences between isoforms. This is a missed opportunity as cancer related isoform variants may serve as biomarkers or therapeutic targets. In order to detect tumor and subgroup specific isoforms, we constructed a tumor-specific protein database using publicly available RNA-seq data from 167 MBs. The spectral data were searched against this customized database and differentially expressed isoforms were identified (Methods). There were relatively few genes with isoforms in different protein groups (36 genes in 72 protein groups); of these, 23 protein groups were significantly differentially expressed (Kruskal-Wallis rank-sum test *p* < 0.05, Additional file 9: TableS4). The most significant were isoforms of the genes CALD1, HMGA1, TMP4, SPTAN1, MCM3, and EEF1D (Fig. 5 and Additional file 10: Figure S6). Caldesmon 1 (CALD1) encodes a calmodulin- and actin-binding protein that plays an essential role in the regulation of smooth muscle and non-muscle contraction. The CALD1 gene consists of at least 15 exons and gives rise to two major classes of isoforms: high molecular weight caldesmon (*h*-CaD) and low molecular weight caldesmon (*l*-CaD) isoforms. *l*-CaD comprises four different splicing variants: WI-38 *l*-CaDs I and II, and Hela *l*-CaDs I and II [25, 26, 30, 52] (Fig. 5a). *h*-CaDs are restricted to fully differentiated smooth muscle cells and regulate muscle tone [26]. *l*-CaDs are ubiquitously expressed in various cells including dedifferentiated SMCs and they play a role in the regulation of cell contractility, adhesion-dependent signaling, cytoskeletal organization, granule movement, hormone secretion and the reorganization of microfilaments during mitosis [6, 26, 75]. We detected WI-38 *l*-CaD II (Protein group A), *h*-CaD and WI-38 *l*-CaD I (Protein group B) isoforms in all meduloblastoma subgroups, but with higher expression in WNT tumors. Interestingly, the HeLa-type caldesmon (protein Group C) were only detected in the WNT subgroup (Fig. 5a). Analysis of mRNA expression of CALD1 isoforms in RNA-seq of 167 tumors confirm these results; the highest expression was found for HeLa *l*-CaD II (transcript NM_033140), being highly expressed in the WNT subgroup (Additional file 11: Figure S7c). Analysis of publicly available H3K27 acetylation marks (a mark of active transcription) in MB tumors [37] validated that the HeLa-type caldesmon isoforms are regulated at the transcriptional level, not due to an alternative splicing mechanism, using an alternative promoter which is especially active in the WNT subgroup compared to the other MB subgroups (Additional file 11: Figure S7b). Interestingly, HeLa-type Caldesmon is located in tumor blood vessels and implicated in the neovascularization of glioma and other tumors [79, 80]. These results are consistent with the observation that WNT tumors stimulate more neo-vascularization compared to the other subgroups [54]. These results together suggest a role for the HeLa *l*-CaD II isoform in MB angiogenesis.

Another interesting set of isoforms are from the high mobility group AT-hook 1 (HMGA1) gene. We detected significantly higher expression of HMGA1 isoforms a and b in Group 3 MB (Fig. 5b). HMGA1 is a DNA binding protein commonly overexpressed in human cancers [1, 2, 8, 10, 56]. Previous studies have reported that HMGA1 is expressed in MB tumors and cell lines where it controls growth, migration and invasion [34, 36]. Here we detected both HMGA1 isoforms significantly expressed in Group 3 MB tumors (Kruskal-Wallis rank-sum test *p*-value = 0.00072 and 0.0178 for isoforms a and b respectively) (Fig. 5b). A significant positive correlation between HMGA1 mRNA and protein was also found (Spearman correlation coefficient = 0.478, Additional file 2: Table S2). These findings were confirmed by RNA-seq (Additional file 12: Figure S8a). Additionally, western blot analysis of the four medulloblastoma groups showed the two HMGA1 isoforms expressed at higher levels in group 3 medulloblastoma (Additional file 12: Figure S8b). Interestingly, the level of total HMGA1 expression is also correlated with poor survival in Group 3 patients (Additional file 12: Figure S8c). Finally, we have correlated HMGA1 expression with MYC expression (Spearman correlation coefficient 0.83) in medulloblastoma group 3 tumors (Additional file 12: Figure S8d), as previously described [73]. These results highlight the potential of proteomics to identify medulloblastoma specific *translated* isoforms and thereby provide biological insights unavailable from genomic data alone.

### Proteome based functional network analysis demonstrates the centrality of the MYC program

**Fig. 6:**
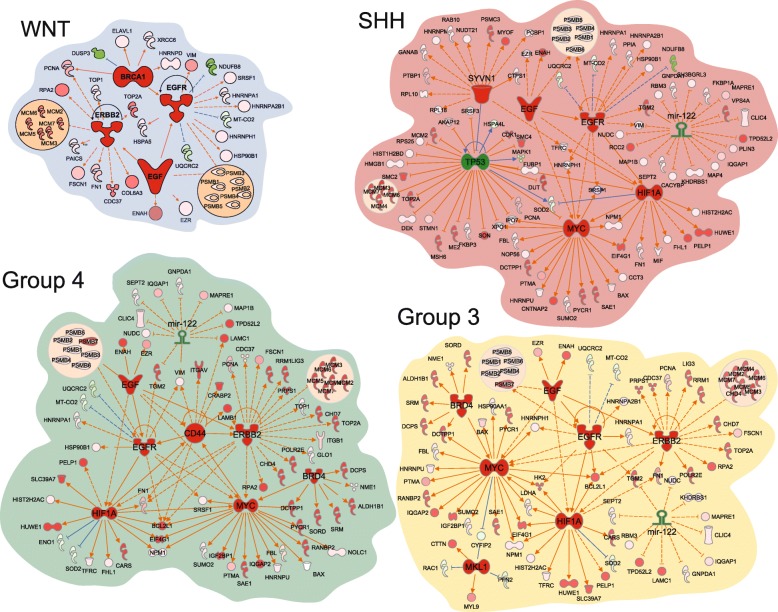
Medulloblastoma subgroup specific upstream regulators. Top upstream regulators predicted by Ingenuity pathway analysis from downstream proteins differentially expressed by subgroup. Upstream regulators are predicted to be active if colored red and inhibited if colored green

In order to identify proteins that are enriched in each of the four medulloblastoma genomic subgroups, protein quantities were determined relative to control cerebellum and then compared between the subgroups (Additional file 13: Table S5 and Additional file 14: TableS6). To analyze the potential for these differentially abundant proteins to inform about subgroup specific biology, we performed Ingenuity Pathway Analysis (IPA) (QIAGEN Inc., https://www.qiagenbioinformatics.com/products/ingenuity-pathway-analysis) [33] using as input the lists of proteins enriched for each subgroup. In this way, we sought to compare protein networks between genomic subgroups using IPA to predict the upstream regulators of the differentially expressed proteins (Additional file 15: Table S7). Notable findings include regulators with shared roles in multiple subgroups: the receptor tyrosine kinases EGFR in all subgroups and ERBB2 in WNT, group 3 and group4; the oncoproteins HIF-1α and MYC in SHH, group 3 and group 4 tumors; the transcriptional activator BRD4 in group 3 and group 4 tumors; and the tumor suppressor mir-122 in group 3, group 4 and SHH tumors (Fig. 6). HIF-1α has been implicated in the maintenance of Notch signaling resulting in the maintenance of neoplastic neural stem cell cells [55, 65]. Transactivated by HIF-1α, MYC is a prominent biological determinant in SHH (MYCN) and group 3 (MYCC) medulloblastoma but has not been widely implicated in group 4 biology, although MYCN amplifications are infrequently observed [48]. BRD4 facilitates MYC-mediated transcriptional activation and as such has been explored as a potential therapeutic target in MYC driven medulloblastoma [3, 69]. Linked to the HIF-1α/MYC/BRD4 axis via HIF1α are the ErbB family members EGFR and ERBB2. ERBB2 has been found to be expressed in a large proportion of medulloblastoma and to be prognostic, however attempts to target it therapeutically have not been successful in the relapsed setting [17, 22]. EGFR is not as well studied in medulloblastoma though there is data to support a synergism between EGFR and Hedgehog signaling in SHH tumors resulting in stabilization of the Gli1 protein [23]. mir-122 is a tumor suppressor that is directly inhibited by MYCC and that, in turn, represses MYCC via its repression of E2f1 and Tfdp2 [71]. It has a well-established role in hepatocellular carcinoma (HCC) where it is down-regulated. Mir-122 knock-out mouse models form HCC and restoration of its expression inhibits tumor development [47]. These data support investigation of mir-122’s role in medulloblastoma.

A potentially confounding issue with normalizing protein quantities back to cerebellum is the tendency to overemphasize proteins associated with cellular proliferation rather than individual subgroup biology. Despite that concern, we also found subgroup restricted upstream regulators including the inhibitory axis of SYNV1-p53 in SHH [76]. This is noteworthy as SHH is the subgroup in which the majority of p53 mutations occur [48]. We also identify the cell adhesion regulator CD44 in group 4 [46], the tumor suppressor BRCA1 in WNT and the anti-apoptotic MKL1 in group 3 tumors. CD44 is a cancer stem cell marker that plays a role in tumor metastasis and progression while regulating multiple signaling networks depending upon the isoforms expressed [51]. Wild-type BRCA1 has been demonstrated to increase the nuclear form of beta-catenin, thereby enhancing the downstream activity of the WNT pathway [35]. MKL1 is part of the RBM15-MKL1 fusion resulting in activation of a Notch pathway transcriptional activator giving rise to acute megakaryoblastic leukemia [43]. It is also a transcriptional modulator in its own right associated with proliferation and invasion in lung and breast cancer [9]. Thus, the top upstream regulators predicted by IPA based on the differentially abundant proteome constitute proteins with known roles in cancer.

Next, IPA assembled the most significant interaction pathways based upon the degree of representation of pathway molecules by differentially expressed proteins (Additional file 16: Figure S9, Additional file 17: Table S8). Pathways previously reported to be important in medulloblastoma were identified including the Ephrin B, mTOR, and integrin signaling pathways [4, 13, 15, 42, 74]. Elements of DNA damage repair were also represented including G2-M checkpoint control and ATM signaling. Metabolism was highlighted by the TCA cycle and oxidative phosphorylation pathways. In keeping with the focus of this report is the prominence of protein synthesis and regulation pathways including the mTOR signaling, protein ubiquitination, EIF2 and EIF4 pathways. In order to demonstrate the potential of proteomics to identify translational opportunities, we chose to validate the importance of EIF4F cap-dependent protein translation to medulloblastoma cell survival.

### EIF4F pharmacologic inhibition

**Fig. 7:**
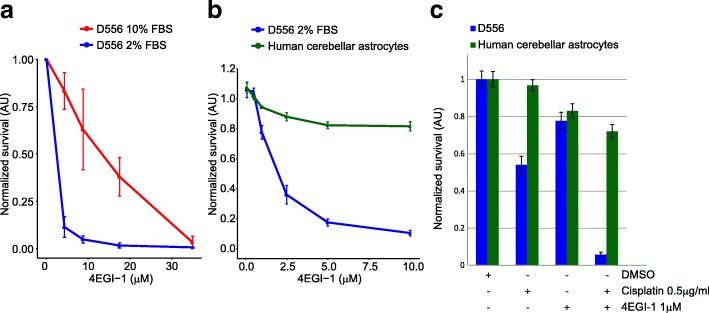
EIF4F inhibitors reduce medulloblastoma cell viability. D556 at normal (10%FBS) and nutrient deprivation (2% FBS) conditions (**a**) and D556 and primary human cerebellar astrocytes cells (**b**) were treated with the EIF4F inhibitor 4EGI-1 for 72 h at the indicated concentrations. The 4EGI-1 toxic concentration in medulloblastoma D556 cells is considerably lower than in normal cerebellar astrocytes. Error bars indicate the standard deviation. **c** Treatment of D556 and primary human cerebellar astrocytes cells with 4EGI-1 in combination with cisplatin

Cancer cells proliferate despite conditions of stress induced by hypoxia and nutrient deprivation. Under stress conditions, the EIF2 pathway is downregulated and the protein translation necessary for cell proliferation is maintained through cap-dependent EIF4F complex translation [64]. Reliance upon robust protein translation has been found across many types of cancer and inhibition of the eIF4F complex is an active area of therapeutic development [38, 62]. We treated 3 different medulloblastoma cell lines (MB002, MB004, D556) with 2 different compounds (4EGI-1 and 4E1RCat) that inhibit the association of eIF4E and eIF4G, thus blocking the formation of the eIF4F complex. We found concentration dependent cell death for all cell lines using both compounds (Additional file 18: Figure S10a). In order to approximate the cellular stress inherent in the tumor microenvironment that gives rise to EIF4F dependent protein translation, we compared sensitivity to the EIF4F inhibitors under modest nutrient deprivation using 2% vs. 10% FCS and found enhanced sensitivity at reduced inhibitor doses (Fig. 7a and Additional file 18: Figure S10b). Next, we treated medulloblastoma and normal human HA-C astrocytes in 2% FCS with toxic concentrations of both compounds and observed a significant disparity in cell death between normal and tumor cells (Fig. 7b and Additional file 18: Figure S10c). Given the infeasibility of obtaining medulloblastoma cells of origin, astrocytes were chosen as they are the most abundant proliferative cell type in the CNS. Treatment with the EIF4F inhibitor 4EGI-1significantly increased the efficacy of cisplatin chemotherapy, a cornerstone of conventional medulloblastoma treatment, while sparing normal astrocytes (Student’s T-test *p* < 0.002, Fig. 7c). These data establish the reliance of medulloblastoma cells in vitro upon cap-dependent translation, a sensitivity that is not shared by normal human astrocytes and supports the further pre-clinical exploration of EIF4F inhibition as a treatment approach with a high therapeutic index.

## Discussion

Genomic characterization of tumors using large sample sets has yielded an unprecedented ability to discriminate subclasses of disease based upon transcriptional programs. While mutations have proven to be the most actionable of the genomic aberrations, pediatric cancers in general have very low mutational burdens. The broad and rich datasets yielded by genomic platforms are ideal for developing a deep understanding of what divides diseases into subgroups. However this endeavor, by its very nature, handicaps efforts to translate genomic findings into therapeutic tools for two reasons. First, focusing on the characteristics that segregate tumors from one another necessarily yields ever finer distinctions. Targeting these differences may yield precise tools that nevertheless fail to address the foundational biology essential to cancer cell survival. Secondly, creating ever smaller patient groups makes it unlikely that industry will create therapeutic agents for them or that subject accrual will allow the testing of any such agent. In contrast, proteomics measures the molecules most proximate to cellular phenotype and downstream of the many regulatory processes that govern the transition from genomic instruction to gene product. As such, it offers the potential to identify those aspects of cancer biology shared between tumor subgroups and indeed even different cancer types.

Quantitative proteomics is a comparative technology. When applied to cancer, the best comparison is to the cell type of origin for that cancer. If the cell of origin is unknown or of a developmental state rendering it unavailable for study, the choice of comparison becomes more contextual. In this study, we chose to compare tumors to normal cerebellum in order to provide a standard normalization and allow for relative comparisons between subgroups. In this way, proteins that were significantly different in more than one subgroup, and thus likely contributing to the biology of those subgroups, would not be lost due to a lack of a difference between those subgroups. We felt this approach was more likely to yield biology common across medulloblastoma and thus more favorable for translation. An alternative approach would have been to perform pairwise comparisons between all subgroups, but such an analysis would have yielded little meaningful biology given the size of the sample set, breadth of protein coverage and number of possible comparisons. Future studies may better apply such an approach, particularly when technological advances enabling higher throughput make larger sample sets more feasible.

Our findings illustrate the unique advantages of proteomics that are both complementary to, and exclusive of genomic output. For example, the observation that there is a relatively poor correlation between transcript and protein abundance provides an example of how proteomics can help clarify which gene expression differences have the potential to influence cellular phenotype. Chromosomal copy number alterations can involve many genes, yet proteomics can help to refine which of those gene products is most affected by the change in gene dose. For example, we found elevated levels of the KPNB1 and ARHGDIA proteins correlating with gained chromosome 17q yet these genes are not correspondingly elevated at the level of the transcript. Thus proteomics allows for the identification and, by virtue of their translation, prioritization of gene products that are candidates to generate phenotype from CNAs. Proteomics, when coupled with RNA-seq data, can also verify the importance of context specific isoform switches by confirming their translation and abundance. For example, we identify translated isoforms of 21 proteins that are restricted to one or more subgroups, thus demonstrating a mechanism beyond genomics driving subgroup biology. Another subgroup constrained protein we have confirmed is HMGA1, a stem cell phenotype regulator that is both a MYC target gene and a regulator of MYC [59, 73]. MYC has protean transformational effects upon cells with cancer specific patterns. In this case, proteomic investigation has highlighted the association of this MYC-HMGA1 effect with group 3 medulloblastoma. Lastly, pathway analysis can be used to predict prominent biological networks from proteomic data. In contrast with genomic networks, such predictions more directly represent cellular function such as the EIF4F pathway identified here. In addition to biological discovery, proteomics offers expanded translational opportunities owing to the fact that tumor proteins are the most accessible pharmaceutical targets, constitute cancer specific antigens for immunotherapy applications, and provide potential biomarkers. Taken together, these advantages demonstrate the necessity of integrating quantitative proteomic discovery data into large scale ‘omic’ based attempts to understand cancer biology.

## Materials and methods

### Clinical samples

The tumor samples used in this manuscript are from Children’s National Health System (Rood Lab, Washington DC, United States), German Cancer Research Center (DKFZ, Pfister Lab, Heidelberg, Germany), Hospital for Sick Children (Taylor Lab, Toronto Canada). The medulloblastoma samples have been classified using the methylation profiling classifier from MolecularNeuropathology.org. Normal cerebellum samples are from the University of Maryland Brain and Tissue Bank. Tissue sample information is listed in Additional file 1: Table S1.

### Cell culture and EIF4E inhibitor treatments

MB002 and MB004 were gifts from Y.J. Cho (Oregon Health & Science University, Portland, OR, United States). R002, R026 and R032 were gifts from A. Moore and W. Ingram (Queensland Children’s Tumour Bank, Brisbane, Australia). DAOY, D556 and D283 cell lines were maintained in Eagle’s Minimum Essential Medium (ATCC) supplemented with 10% fetal bovine serum (ATCC) and 100 U/mL penicillin and streptomycin (ATCC). All established cell lines were verified with STR analysis (GRCF at Johns Hopkins). R026, R032 and R060 cell lines were maintained in Dulbecco’s Modified Eagle’s Medium/Ham’s Nutrient Mixture F12 (ATCC) without HEPES supplemented with 10% fetal bovine serum (ATCC), Glutamine (ATCC) and 100 U/mL penicillin and streptomycin (ATCC). MB002 and MB004 cells were maintained in culture media with 1:1 Dulbecco modified Eagle medium (Gibco) and neural stem cell media (Gibco) supplemented with non-essential aminoacids (Gibco), Sodium Pyruvate (Gibco), HEPES, GlutaMax (Gibco), B27 (Gibco), EGF (Gibco), FGF (Millipore), Heparin (Stem Cell),LIF (Millipore), 10% fetal bovine serum (ATCC) and 100 U/mL penicillin and streptomycin (ATCC). Primary human normal astrocytes-Cerebellar were acquired from ScienCell and maintained in Astrocyte medium (ScienCell) supplemented with Fetal Bovine Serum (ScienCell), astrocyte grow supplement (ScienCell) and penicillin/streptomycin (ScienCell). All cells lines were maintained at 37 °C with 5% CO2 in a 95% humidified atmosphere.

For EIF4E inhibitor treatment, 1000 cells were plated in a 96 well plate in triplicates with dilutions of the EIF4E inhibitors, 4EGI-1(Selleckchem) and 4E1RCat (Selleckchem), and the chemotherapy agent cisplatin (TOCRIS Bioscience). DMSO was used as control. Cells were serum starved for 24 h before treatment and seventy-two hours after treatment cell viability was analyzed using the CellTuter-Glo®2.0 Assay kit (Promega) according to the manufacture recommendations. Experiments were repeated in triplicate.

### SILAC proteomic analysis

We created a pooled super-SILAC reference atlas, termed the Labeled Atlas of Medulloblastoma Proteins (LAMP), by combining equal amounts of isotopically labeled proteins from 8 primary and established cell lines chosen to represent the breadth of medulloblastoma across the four genomic subgroups (DAOY, D556, D283, R026, R032, R060, MB002, and MB004). Super-SILAC refers to the use of multiple cell lines to create the reference atlas as opposed to just one. Briefly, cell lines are passaged in growth media that has been depleted of lysine and arginine. To this media, lysine and arginine incorporating 6 carbon isotopes (_13_C_6_) was added. The proteins made by these cells incorporate the isotopic amino acids creating a predictable mass increase. Serial aliquots were tested over time until isotopically labeled proteins exceeded 95% of the total. Mass spectrometry of the LAMP itself identified 4903 proteins.

We spiked in the LAMP at a ratio of 1:1 based upon BCA protein quantitation into protein lysates from 36 medulloblastoma tumors and 5 control cerebellum samples. 100 μg of protein from each sample was run in triplicate using 1-D SDS PAGE fractionation, cut into 32 bands and in-gel trypsin digested. Concentrated peptides from each band were injected via an autosampler (6uL) and loaded onto a Symmetry C18 trap column (5 mm, 300 μm i.d. × 23 mm, Waters) for 10 min at a flow rate of 10 μL/min, 100% A. The mobile phases consisted of water with 0.1% formic acid (A) and 90% acetonitrile (B). The sample was subsequently separated by a C18 reverse-phase column (3 μm, 200A, 100 mm × 15 cm, Magic C18, Michrom Bioresources) at a flow rate of 300 nL/min using an Eksigent nano-HPLC system (Dublin, CA). A 65 min linear gradient from 5 to 60% B was employed. Eluted peptides were introduced into the mass spectrometer via Michrom Bioresources CaptiveSpray. The spray voltage was set at 1.4 kV and the heated capillary at 200 °C. The LTQ-Orbitrap-XL (ThermoFisherScientific) was operated in data-dependent mode with dynamic exclusion in which one cycle of experiments consisted of a full-MS in the Orbitrap (300–2000 m/z) survey scan in profile mode, resolution 30,000 and five subsequent MS/MS scans in the LTQ of the most intense peaks in centroid mode using collision-induced dissociation with the collision gas (helium) and normalized collision energy value set at 35%.

For protein identification and quantification we used MaxQuant version 1.5.3.30 software developed by Max Planck Institute of Biochemistry (http://www.biochem.mpg.de/5111795/maxquant). Mass spectral data were uploaded into MaxQuant software. Files from each lane were searched against the forward and reverse Uniprot human database (UniProt release 2016_05 with 20,201 entries) for partially tryptic peptides allowing one missed cleavage, and possible modification of oxidized methionine (15.99492 Da) and heavy arginine (6.0201 Da) and heavy lysine (8.0142 Da). MaxQuant uses the Andromeda search engine. Mass tolerances were set at +/− 20 ppm for first peptide search and +/− 4.5 ppm for main peptide search, with intensity threshold of 500. Data were filtered based on a 5% protein false discovery rate. All the bands from each lane were summed as one sample in the analysis.

### Microarray gene expression analysis

To generate gene expression profiling data, 19 tumor and 5 control specimens were homogenized in Trizol followed by phase separation of nucleic acids with chloroform. RNA was extracted using Picopure RNA isolation kit (Arcturus Bioscience, Mountain View, CA). DNA was removed by treating columns with RNase Free DNase (Qiagen, Valencia, CA). RNA integrity and concentration were quantified using a 2100 Bioanalyzer (Agilent Technologies, Santa Clara, CA). The GeneChip WT Plus kit (Affymetrix, Santa Clara, CA) was used for cRNA synthesis from total of 250 ng of RNA. cRNA was hybridized to GeneChip Human Gene 2.0 ST Array (Affymetrix, Santa Clara, CA) and probe fluorescence intensity was detected with GeneChip® System 3000Dx v.2 (Affymetrix, Santa Clara, CA). Gene expression profiles for the rest of the tumors (16) were previously generated;7 using the GeneChip™ Human Genome U133A 2.0 Array and 9 using the Human Gene 1.1 ST Array (GEO accession number GSE37385 and GSM324067 respectively) (Additional file 1: Table S1). Expression data for each Chip type were analyzed independently in the R environment (v3.4.1) (https://www.r-project.org) with the oligo package (v1.36.1). Differentially expressed genes for each subgroup versus cerebellum were calculated using the limma R-package (v3.28.21).

### Evaluating mRNA-protein correlation

*mRNA and protein expression values*. For mRNA, expression data values (log2) from every chip type were merged and corrected for potential chip type effect applying an Empirical Bayes method using the combat function from the sva R package (v3.20.0). For proteins, we used the ratio of heavy over light (H/L) values as described above.

#### Number of overlapping genes in proteomic and mRNA data sets

The proteomic data set used for mRNA-protein correlation (35 tumors, Additional file 1: Table S1) included a total of 2846 gene products quantified in at least 1 tumor; 2430 of these were present in the mRNA expression dataset. To compare mRNA and protein variation across tumors, we focused on 1240 genes with quantified protein in at least one third of the tumors. For subgroup specific analysis, we used genes with quantified protein in at least one half of the tumors.

#### Correlation between mRNA and protein variation

We first calculated the Spearman correlation coefficient between the mRNA expression values (log2) and the protein H/L ratio for each of the 1240 genes. Then, *p*-values corresponding to the coefficients were calculated with < 0.05 considered significant. The same approach was applied to each subgroup. Correlation differences among the four subgroups were evaluated based on the Kruskal-Wallis rank-sum test and differences between Group 4 and the rest was evaluated based on a two-sided Wilcoxon rank-sum test.

#### KEGG pathway analysis

Based on Spearman correlation coefficients, we separated the highly correlated mRNA-protein pairs (Quartile 3) from those with low correlation (Quartile 1). Next, KEGG pathway analysis using the DAVID Bioinformatics Database (DAVID Bioinformatics Resources, http://david.abcc.ncifcrf.gov/) was performed with the protein identifier of genes in each group to find enriched biological processes. Pathways with a Bonferroni corrected p-value < 0.01 in at least one group were selected.

#### mRNA-protein correlation versus molecular stability

To analyze the relationship between mRNA-protein correlation and stability, we used mRNA and protein half-life data from mouse fibroblast cell line [58]. Only genes (456) in common between both our datasets and the mouse fibroblast dataset were included in the analysis. mRNA or proteins with half-life values higher than the upper quartile (Q3) were considered stable and those with half-lives in the lowest quartile (Q1) were considered unstable. Correlation differences between the 2 categories were calculated based on two-sided Wilcoxon rank-sum test.

### Genome wide methylation analysis

DNA (500 ng) was obtained from 35 tumor and 5 control cerebellum tissue lysates using the Gentra Puregene DNA extraction kit (Quiagen, Valencia, CA) (Additional file 1: Table S1). DNA was prepared for methylation analysis via bisulphate conversion using EZ DNA Methylation-Gold kit (Zymo Research, Irvine, CA). Bisulphite-converted DNA was denatured and neutralized. After amplification via PCR, DNA was fragmented and hybridized onto the Infinium Methylation EPIC BeadChip (Illumina, San Diego, CA). Raw data files (.idat) generated by the Illumina iScan system were processed in the R statistical environment (v3.3.1) (https://www.r-project.org) using minfi (v 1.22.1) and ChAMP (v2.4.1) packages. Probes were removed if their *p*-value was above 0.01 in one or more samples. Additionally, we filtered out probes with a beadcount lower than 3 in at least 5% of samples. Finally, we removed probes on the sex chromosomes as well as those located on or close to known single nucleotide polymorphism (SNP). We retained a total of 749,678 probes for the analysis. The data was normalized using the BMIQ method. Differentially methylated regions (DMRs) were calculated for each subgroup versus cerebellum using ChAMP R-package with the DMRcate method [53].

### Methylation array copy number analysis

Copy number segmentation was performed from the genome wide methylation arrays using the conumee R-package (v1.8.0) using normal cerebellum as diploid controls. Segmented copy number estimates were processed for input with GISTIC2 [44] using the default parameters. The copy number by genes was used for the correlation analysis. Significant broad chromosome arm lesions were evaluated across all samples without separating them between subgroups (q-value < 0.1).

### Effect of methylation and copy number on protein expression

#### Correlation of methylation status and gene/protein expression

Differentially methylated regions (DMRs) were calculated comparing each subgroup to cerebellum using ChAMP R-package with the DMRcate [53] method. Each DMR was assigned to a gene according to their coordinates in the hg19 reference genome. Differentially expressed proteins or genes (up- or down-regulated) were correlated with their associated absolute DMR values (gain or loss). Odds ratios were calculated for each subgroup using Fisher’s exact method.

#### Correlation of copy number alteration and gene/protein expression

The matched CNA (Copy Number alteration), protein and mRNA abundance measurements from 32 tumors (Additional file 1: Table S1) were used to study the impact of CNA on gene and protein expression. We focused on 1148 genes with quantified proteins in at least 33% of the tumors that were also included in the CNA and mRNA datasets. For each of those genes, we calculated the Spearman correlation coefficient between CNA and mRNA/protein measurements. Significant calls were made based on a *p*-value cutoff of 0.05.

### NMF clustering

NMF clustering was performed from the proteomic ratios H/L using the NMF R-package (v0.20.0) [19]. For NMF clustering we used proteins quantified in at least 40% (1158 proteins) of the samples in the dataset. The missing ratio L/H values (NAs) were replaced by the lowest value found in the dataset (0.05) to approximate the lower limit of detection. We used the NMF algorithm brunet [5] with 5000 runs for each factorization rank *k* between 2 and 10 using the default seed method. The same NMF settings were applied to the transcript abundance of each of the genes coding for the proteins used in the proteomic classification.

### Differentially expressed proteins

Proteins from each medulloblastoma subgroup were compared to control cerebellum in a pairwise fashion using the R environment (v3.4.1) with statistical confidence measured using the Wilcoxon rank-sum test at a p-value cut-off of 0.05. Proteins were also required to be present in at least half of the total replicates of both subgroups in a comparison. To these differentially abundant proteins were added the subgroup restricted proteins, those that were present in at least half of the samples of one subgroup and absent in the remaining samples.

### Pathway analysis

Pathway analysis of differentially expressed proteins was performed using Ingenuity Pathway Analysis (IPA) v. 01–04. Lists of differentially expressed proteins and their fold changes for each genomic subgroup, compared to control cerebellum, were imported into IPA. For this analysis, we removed from consideration the 2 tumor samples that clustered separately given their distinctiveness. Proteins that were present in one group and absent in another were arbitrarily assigned a fold change value equal to the average fold change of quantifiable proteins +/− two standard deviations. Core expression analysis was created for each of the lists considering direct and indirect relationships using Ingenuity Knowledge Base genes to calculate *p*-values. Top pathways for each subgroup were then selected based on highest ranked p-values (Additional file 14: Table S6).

### Protein isoform quantification

To create the customized MB specific isoform database, we used the R package customProDB (v1.16) [72]. RNA-seq data from 167 publicly available MB tumors (Datasets: EGAD00001001899, EGAD00001002683, EGAD00001001210, EGAD00001001620, and EGAD00001000328) were mapped to human genome GRCh38/hg38 using STAR(v.2.5.1) [14]. Protein coding transcripts (UCSC RefSeq annotation) were quantified using the calculateRPKM function of the customProDB package. Only protein coding transcripts expressed in at least 5% of the samples and belonging to the top 80% by expression were included in the database. For protein isoform identification and quantification, we used MaxQuant software (v.1.5.3.30). Files from each lane were searched against the MB specific isoforms database for partially tryptic peptides allowing one missed cleavage, and possible modification of oxidized methionine (15.99492 Da), heavy arginine (6.0201 Da) and heavy lysine (8.0142 Da). Mass tolerances were set at +/− 20 ppm for first peptide search and +/− 4.5 ppm for main peptide search, with an intensity threshold of 500. Data were filtered based on a 5% protein false discovery rate. All the bands from each lane were summed as one sample in the analysis. For differential isoform expression, we focused on genes that contain isoforms in more than one protein group. The statistical confidence of the differentially expressed isoforms was measured using the Kruskal-Wallis rank-sum test. Significant calls were made based on a *p*-value cutoff of 0.05 (Additional file 17: Table S8).

### RNA sequencing

RNA-seq data from 167 publicly available MB tumors (Datasets: EGAD00001001899, EGAD00001002683, EGAD00001001210, EGAD00001001620, and EGAD00001000328) were mapped to human genome GRCh38/hg38 using STAR(v.2.5.1) [14]. Genes and isoform expression was quantified using the cufflinks software (v2.2.1) [68] and hg38 UCSC transcript annotation.

### Survival analysis

Overall survival functions were estimated using the Kaplan-Meier method and *p*-values were calculated using the log-rank test. The statistical analysis was performed in the R statistical environment using the R package survival (v2.41–3) and survminer (v0.4.0). Gene expression and overall survival data from 113 Group 3 medulloblastoma tumors were downloaded from a previously published dataset [7] (GEO accession data: GSE85218). Group 3 medulloblastoma tumors were separated into samples with HMGA1 expression greater and less than the median.

### Western immunoblotting

Protein extracts were prepared by lysing tumor tissue cells in RIPA Lysis buffer (Millipore) containing 50 mM Tris–HCl, pH 7.4, 1% Nonidet P-40, 0.25% sodium deoxycholate, 150 mM NaCl, 1 mM EDTA, 1× protease inhibitor cocktail (Roche Applied Science). Tumor lysates were resolved by SDS-PAGE, transferred to a nitrocellulose membrane, and incubated with HMGA1 antibodies (D6A4, Cell Signaling). Protein detection was performed using ECL Western blotting detection reagents (SuperSignal West Dura, Thermo Scientific).

## Conclusions

Quantitative proteomics offers an important dimension beyond genomics, proximate to the molecular disease processes underlying malignancy. Here, we present the largest tissue based Super-SILAC quantitative proteomics study to date demonstrating how proteomics complements genomic platforms to yield a more complete understand of functional tumor biology and identify novel therapeutic targets for medulloblastoma. We conclude that there is a poor correlation between epigenetic features/transcript level and protein abundance. However, proteomics can help distill the most salient genomic features based upon the axiom that a genetic event must be translated to the proteome in order to exert a functional effect upon the cell. Proteomics can also help identify which genes are affected by dosage changes resulting from chromosomal copy number alterations. In concert with RNA-seq, proteomics can identify disease-specific translated splice isoforms as well as novel protein isoforms. While proteomic data can be used to recapitulate the genomic subgroups, the gene products it uses to do so are different than those identified with genomic platforms, thus offering a new cohort of targets for attempted intervention. Indeed, signaling networks built from protein data are more reflective of cell biology and therefore a more robust source of therapeutic targets. Thus proteomics can be used retrospectively to help interpret and filter the wealth of genomic findings as well as prospectively to map out functional cellular biology to be exploited for therapeutic development.

## Additional files

Additional file 1: **Table S1**. Clinical and tumor sample information. (XLSX 12 kb)

Additional file 2: **Table S2**. Correlation between mRNA and Protein abundance. Column names legend. Gene: Gene name symbol, TumorID_Prot: Protein quantification value, TumorID_RNA: mRNA quantification value, corr: spearman correlation coefficient, *p*-value: spearman correlation coefficient *p*-value. (XLSX 2 mb)

Additional file 3: **Figure S1**. Correlation between mRNA and protein abundance by subgroups. a) Frequency distribution plots of mRNA-protein spearman’s correlations for each medulloblastoma subgroup. Positive mRNA-protein correlations were found for 68–80% of mRNA-protein pairs with means between (0.30–0.16) depending on the subgroup. However, just a small proportion of them were significant (10–17%). Group 3 was the subgroup with the highest number of significant positive correlations and the highest mean; in contrast, group 4 had the lowest mean. b) Box-plots depicting the distribution of mRNA-protein Spearman’s correlations by subgroup. *p*-values for differences between the subgroups were calculated with the Kruskal-Wallis rank-sum test. *p*-values indicating the differences between group 4 and the others were calculated with a two-sided Wilcoxon rank sum test. (PDF 706 kb)

Additional file 4: **Figure S2**. Effect of DNA methylation on mRNA and protein abundance. a) Differentially expressed proteins and RNA, and differentially methylated regions (DMR) were calculated for each subgroup compared to control cerebellum. The pie charts indicate the percentage of differentially expressed proteins (upper panel) or transcripts (lower panel) associated with a DMR. b) Correlation between increased/decreased expression of protein (upper panel) and mRNA (lower panel) and the loss/gain of methylation on the associated gene promoter. Odds ratio and *p*-value using Fisher’s exact method were calculated for each subgroup. (PDF 676 kb)

Additional file 5: **Figure S3**. Correlation between copy number, mRNA and protein abundance. a) Frequency distribution plots of Spearman’s correlations for CNA-mRNA (left) and CNA-protein (right). Positive CNA-mRNA and CNA-protein correlations were found in 81 and 69% of the CNA-mRNA-protein trios with a mean 0f 0.12 in both cases. However, only 18% of mRNA-CNA and 13% of mRNA-protein correlations were significant. b) Representation across the genome of significant correlations for CNA-mRNA (upper panel) and CNA-Protein (lower panel). (PDF 2.24 mb)

Additional file 6: **Table S3.** Correlation between CNA and mRNA and Protein abundance. Column names legend. Gene: Gene name symbol, TumorID_Prot: Protein quantification value, TumorID_RNA: mRNA quantification value, TumorID_CN: Copy Number value, corr_Prot_CN: spearman correlation coefficient between protein and copy number, pvalue_Prot_CN: spearman correlation coefficient *p*-value between protein and copy number, corr_RNA_CN: spearman correlation coefficient between mRNA and copy number, pvalue_RNA_CN: spearman correlation coefficient p-value between mRNA and copy number, Cytoband: Gene cytoband, Coord: Gene coordinates in GRch38 reference genome. (XLSX 1.30 mb)

Additional file 7: **Figure S4**. Copy number effect on mRNA and protein abundance on chromosome arm 17q. a) GRB2, LASP1, KPNB1 and ARHGDIA showed significant CNA-protein correlations (*p* < 0.05). The colors depict the range from low (white) to high (green) of copy-number, protein and mRNA abundance. Samples were ranked by copy number at each gene locus. b) ARHGDIA protein was found to be significantly overexpressed in group 4 tumors which frequently harbor 17q gains but not at the mRNA transcript level. Differences among the four subgroups were evaluated based on the Kruskal-Wallisrank-sum test. (PDF 916 kb)

Additional file 8: **Figure S5**. Proteomic subgroup classification recapitulates genomic subgroups using different data elements. Comparison of non-negative matrix factorization consensus clustering between protein and mRNA expression data from 34 primary medulloblastoma and five normal cerebellar tissues. a) The Cophenetic and Silhouette coefficient values for rank *k* between 2 and 10 in mRNA and protein dataset. b) NMF clusters (k = 6) for the same genes coding for the proteins used in the proteomic classification at the mRNA or protein level. Clustering did not improve for other k values 2 through 10 (data not shown). (PDF 994 kb)

Additional file 9: **Table S4.** Differentially expressed isoforms.

Additional file 10: **Figure S6**. Medulloblastoma subgroup specific isoforms. Schematic representation of MCM3, TPM4, SPTAN1 and EEF1D isoforms. Boxplots show the quantification of each protein isoforms group across all medulloblastoma subgroups. *p*-values for differences between subgroups were calculated based on the Kruskal-Wallis rank-sum test. A protein group is defined as the group of isoforms that are indistinguishable due to the position of identified peptides. (PDF 1.13 mb)

Additional file 11: **Figure S7**. Expression of CALD1 isoforms in medulloblastoma tumors. The protein expression level of CALD1 isoforms in medulloblastoma subgroups is confirmed at the epigenetic (H3K27Ac Chip-seq) and mRNA level. a) Schematic representation of CALD1 isoforms. b) H3K27Ac Chip-seq genome tracks in medulloblastoma tumors. Active transcription region marks (H3K27Ac) are observed in the alternative transcription start site for the isoforms HeLa l-CaD I and II correlating with higher expression of these protein isoforms. c) Boxplots representing the mRNA expression levels for CALD1 isoforms. (PDF 2.2 mb)

Additional file 12: **Figure S8**. Expression of HMAG1 isoforms in medulloblastoma tumors. a) Schematic representation of HMGA11 isoforms and Boxplots representing the mRNA expression levels for HMGA1 isoforms. b). Western blot of HMGA1 isoforms in the four medulloblastoma subgroups. Both HMAG1 isoforms are highly expressed in group 3 medulloblastoma. c) Kaplan–Meier survival curve shows that increased levels of HMGA1 are associated with poor survival in Group 3 Medulloblastoma. d) Expression level of HMGA1 is highly correlated with the expression of the oncogene MYC in Group 3 Medulloblastoma. (PDF 1.94 mb)

Additional file 13: **Table S5**. List of Differentially expressed proteins. (XLSX 235 kb)

Additional file 14: **Table S6**. List of present and absent proteins. (XLSX 153 kb)

Additional file 15: **Table S7**. List of upstream regulator for each medulloblastoma subgroup. (XLSX 15.9 kb)

Additional file 16: **Figure S9**. Subgroup representative pathways. Enriched pathways generated with Ingenuity Pathway Analysis software based on lists of differentially quantitated proteins by subgroup normalized to control cerebellum. Each circle plots the *p*-value for a pathway in each medulloblastoma subgroup. (PDF 623 kb)

Additional file 17: **Table S8**. List of pathways representative for each medulloblastoma subgroup. (XLSX 72.6 kb)

Additional file 18: **Figure S10**. EIF4 inhibitor concentration dependent cell death in medulloblastoma cells. a) MB002, MB004 and D556 cells were treated with the EIF4F inhibitors 4E1RCat for 72 h at the indicated concentrations. In all cell lines we found concentration-dependent cell death when treated with EIF4F inhibitors. D556 at normal (10%FBS) and nutrient deprivation (2% FBS) conditions (b) and D556 and primary human cerebellar astrocytes cells (c) were treated with the EIF4F inhibitor 4E1RCat for 72 h at the indicated concentrations. Error bars indicate the standard variation deviation. d) Treatment of D556 and primary human cerebellar astrocytes cells with 4E1RCat in combination with cisplatin at the indicated concentrations. (PDF 975 kb)
