# Supplementary material for: Proteomic analysis of Medulloblastoma reveals functional biology with translational potential
Source: Acta Neuropathol Commun. 2018 Jun 7;6:48. doi: 10.1186/s40478-018-0548-7 (PMC5992829; doi:10.1186/s40478-018-0548-7)
Supplement: Supplementary file 3 — Figure S1. Correlation between mRNA and protein abundance by subgroups. a) Frequency distribution plots of mRNA-protein spearman’s correlations for each medulloblastoma subgroup. Positive mRNA-protein correlations were found for 68–80% of mRNA-protein pairs with means between (0.30–0.16) depending on the subgroup. However, just a small proportion of them were significant (10–17%). Group 3 was the subgroup with the highest number of significant positive correlations and the highest mean; in contrast, group 4 had the lowest mean. b) Box-plots depicting the distribution of mRNA-protein Spearman’s correlations by subgroup. p-values for differences between the subgroups were calculated with the Kruskal-Wallis rank-sum test. p-values indicating the differences between group 4 and the others were calculated with a two-sided Wilcoxon rank sum test. (PDF 706 kb) [file 40478_2018_548_MOESM3_ESM.pdf]

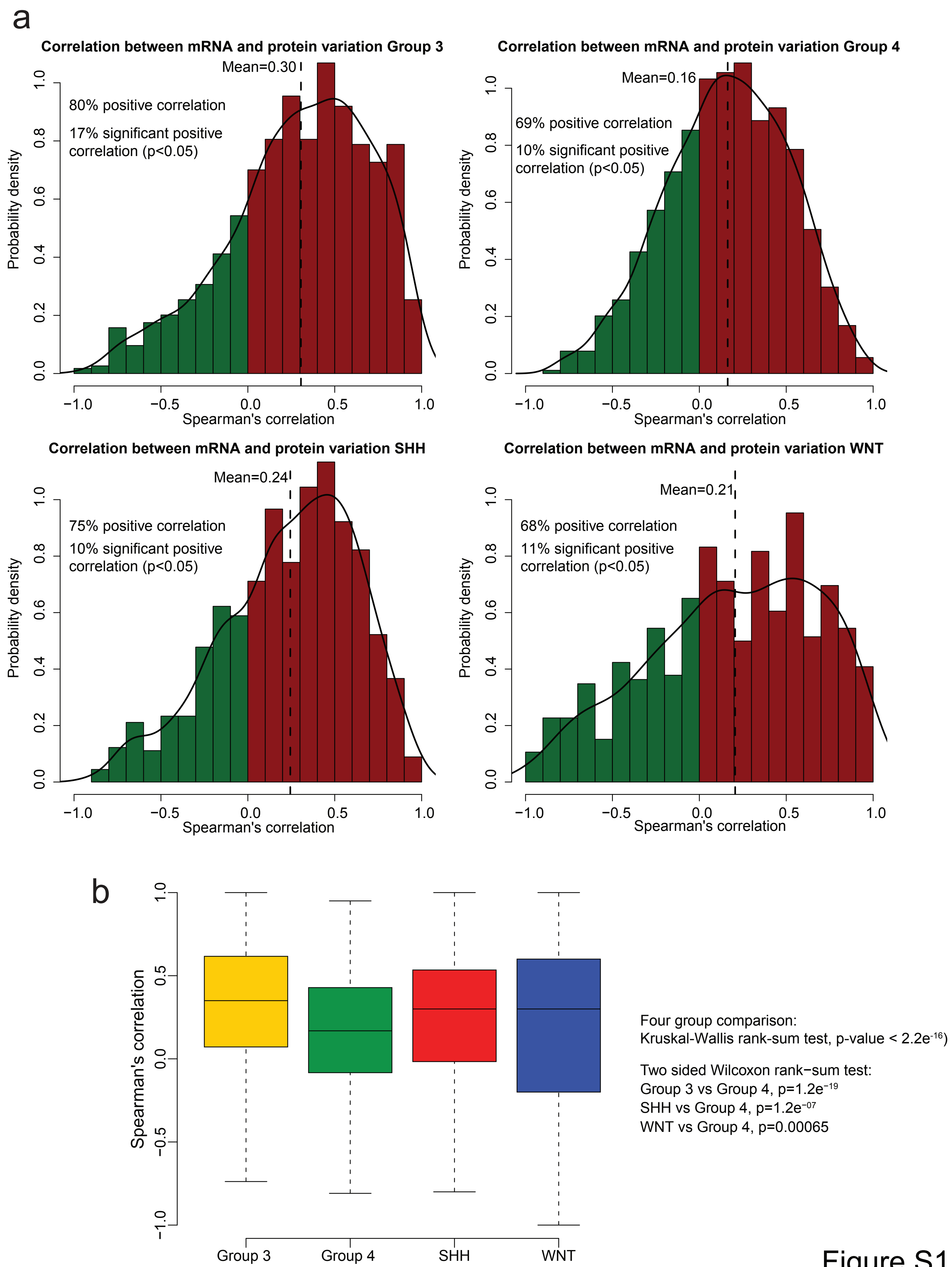
a
Correlation between mRNA and protein variation Group 3
Mean=0.30
80% positive correlation
17% significant positive correlation (p<0.05)
Probability density
Spearman's correlation
Correlation between mRNA and protein variation Group 4
Mean=0.16
69% positive correlation
10% significant positive correlation (p<0.05)
Correlation between mRNA and protein variation SHH
Mean=0.24
75% positive correlation
10% significant positive correlation (p<0.05)
Correlation between mRNA and protein variation WNT
Mean=0.21
68% positive correlation
11% significant positive correlation (p<0.05)
b
Spearman's correlation
Group 3
Group 4
SHH
WNT
Four group comparison:
Kruskal-Wallis rank-sum test, p-value < 2.2e-16)
Two sided Wilcoxon rank−sum test:
Group 3 vs Group 4, p=1.2e-19
SHH vs Group 4, p=1.2e-07
WNT vs Group 4, p=0.00065

Figure S1
