# Supplementary figures and images for: Proteomic analysis of Medulloblastoma reveals functional biology with translational potential
Source: Acta Neuropathol Commun. 2018 Jun 7;6:48. doi: 10.1186/s40478-018-0548-7 (PMC5992829; doi:10.1186/s40478-018-0548-7)

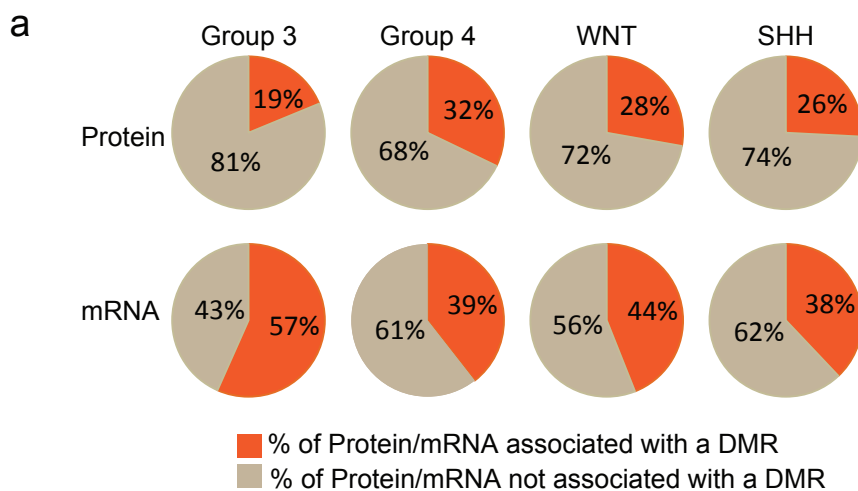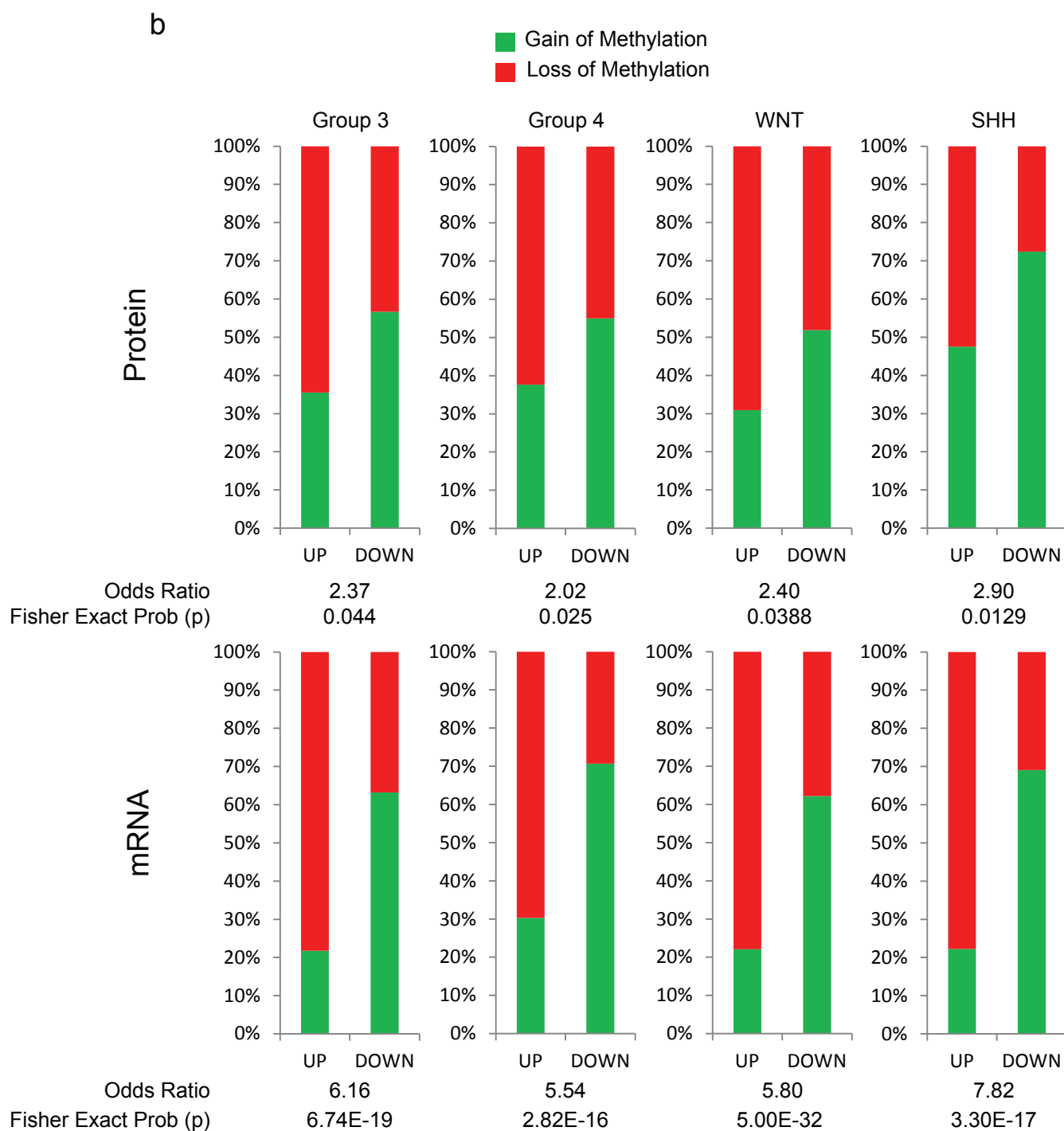

Figure S2

Supplement: Supplementary file 4 — Figure S2. Effect of DNA methylation on mRNA and protein abundance. a) Differentially expressed proteins and RNA, and differentially methylated regions (DMR) were calculated for each subgroup compared to control cerebellum. The pie charts indicate the percentage of differentially expressed proteins (upper panel) or transcripts (lower panel) associated with a DMR. b) Correlation between increased/decreased expression of protein (upper panel) and mRNA (lower panel) and the loss/gain of methylation on the associated gene promoter. Odds ratio and p-value using Fisher’s exact method were calculated for each subgroup. (PDF 676 kb) [file 40478_2018_548_MOESM4_ESM.pdf]

# Correlation between Protein/mRNA and Copy number variation

a

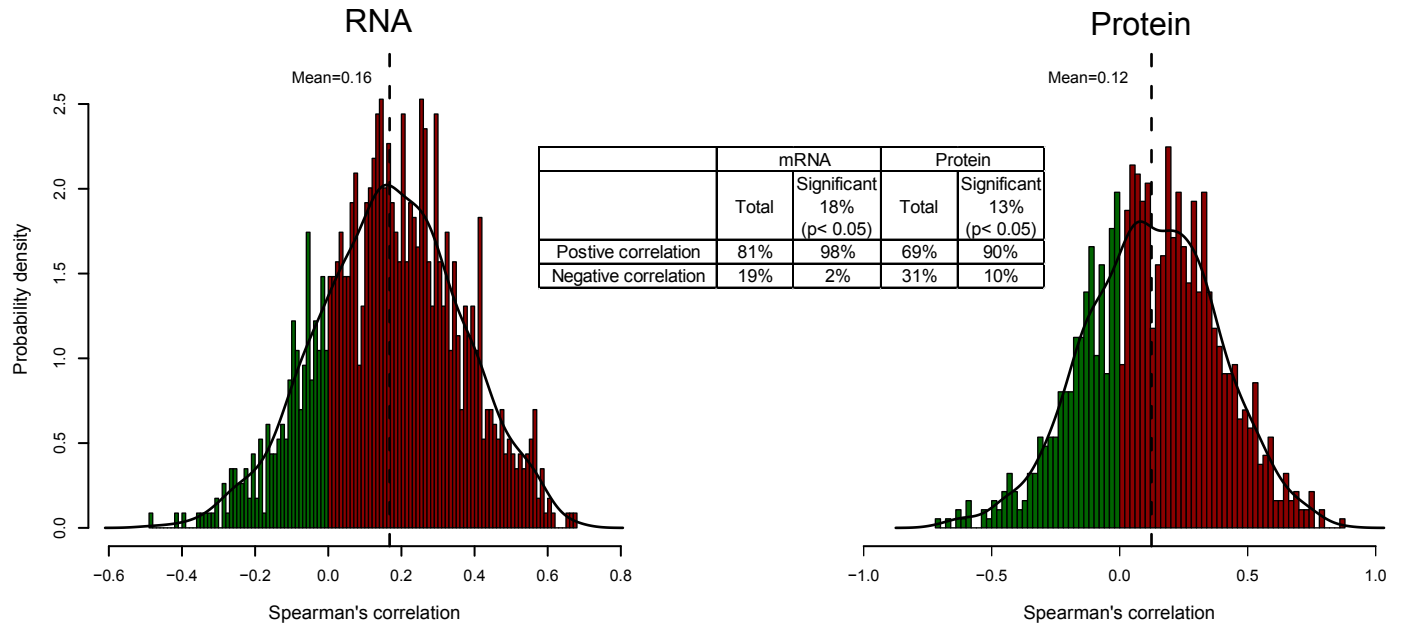

b

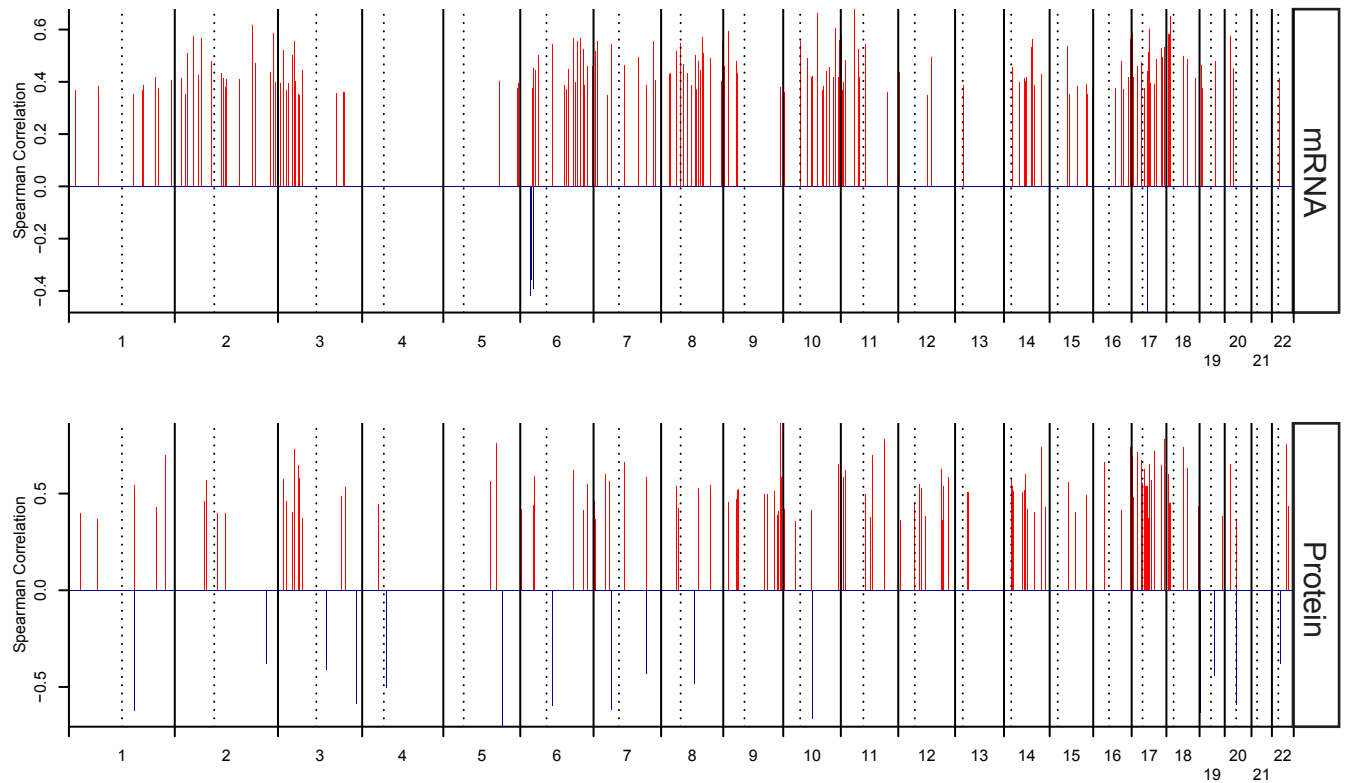

Figure S3

Supplement: Supplementary file 5 — Figure S3. Correlation between copy number, mRNA and protein abundance. a) Frequency distribution plots of Spearman’s correlations for CNA-mRNA (left) and CNA-protein (right). Positive CNA-mRNA and CNA-protein correlations were found in 81 and 69% of the CNA-mRNA-protein trios with a mean 0f 0.12 in both cases. However, only 18% of mRNA-CNA and 13% of mRNA-protein correlations were significant. b) Representation across the genome of significant correlations for CNA-mRNA (upper panel) and CNA-Protein (lower panel). (PDF 2.24 mb) [file 40478_2018_548_MOESM5_ESM.pdf]

a

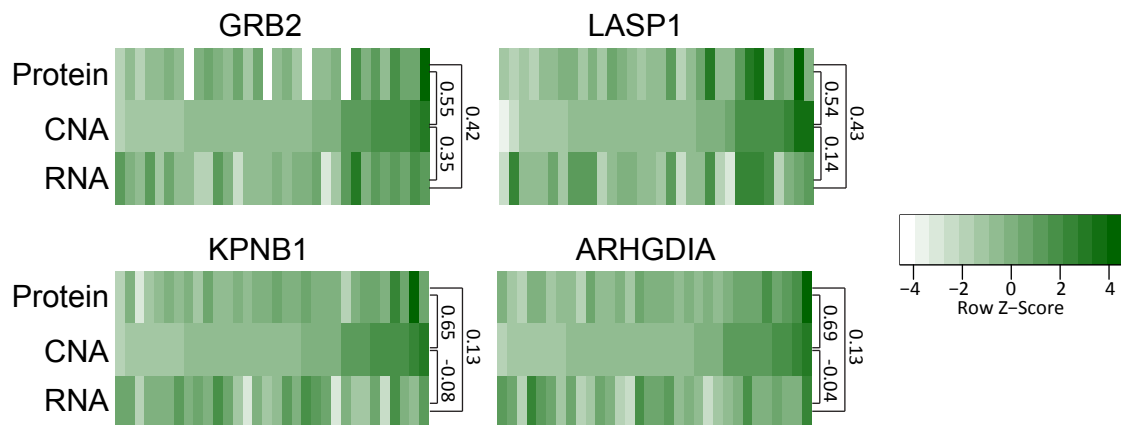

b

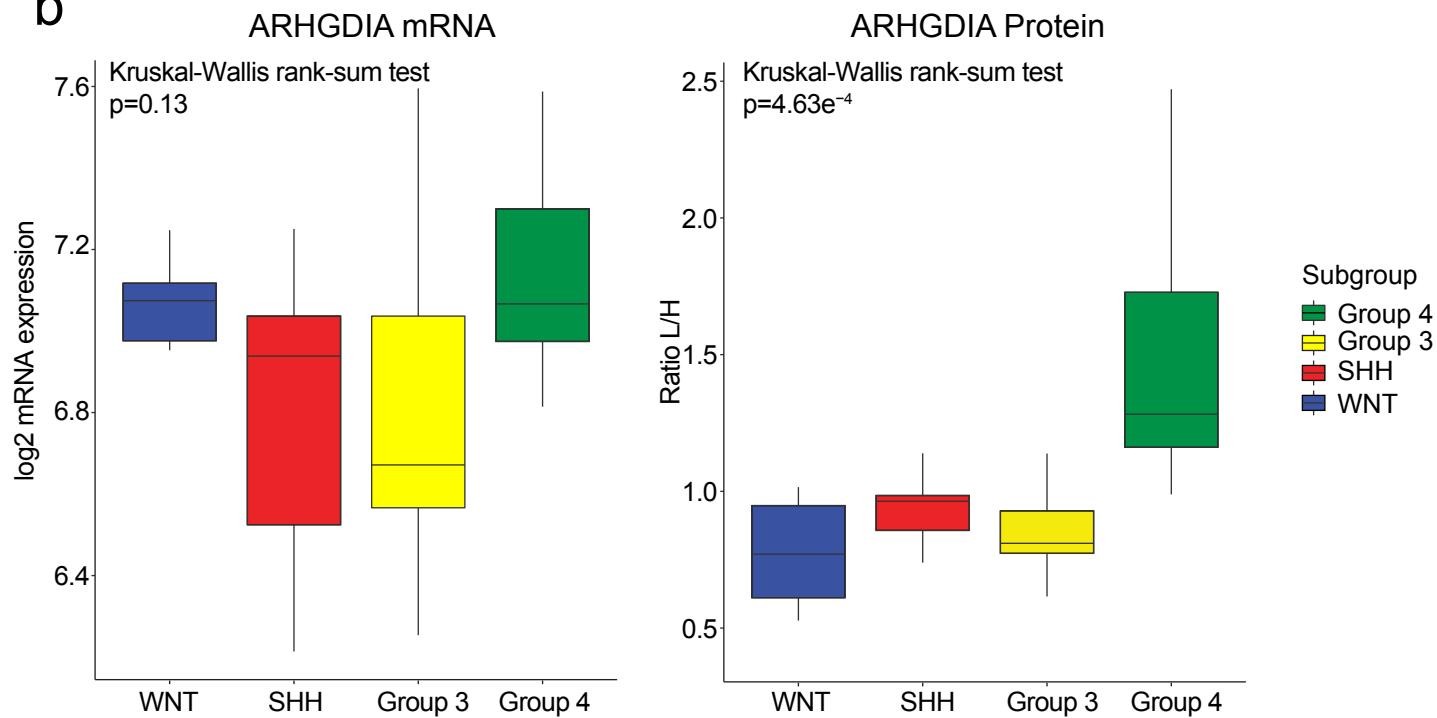

Figure S4

Supplement: Supplementary file 7 — Figure S4. Copy number effect on mRNA and protein abundance on chromosome arm 17q. a) GRB2, LASP1, KPNB1 and ARHGDIA showed significant CNA-protein correlations (p < 0.05). The colors depict the range from low (white) to high (green) of copy-number, protein and mRNA abundance. Samples were ranked by copy number at each gene locus. b) ARHGDIA protein was found to be significantly overexpressed in group 4 tumors which frequently harbor 17q gains but not at the mRNA transcript level. Differences among the four subgroups were evaluated based on the Kruskal-Wallisrank-sum test. (PDF 916 kb) [file 40478_2018_548_MOESM7_ESM.pdf]

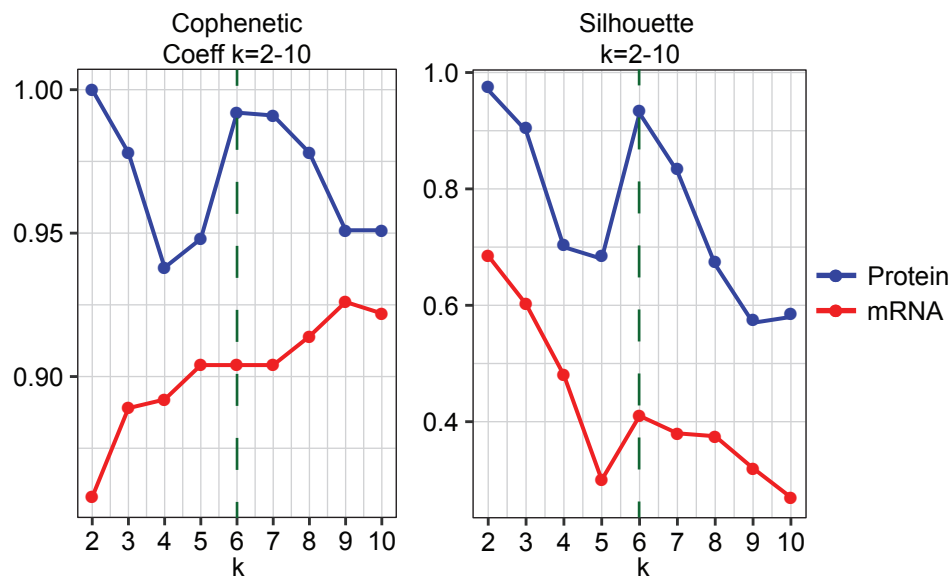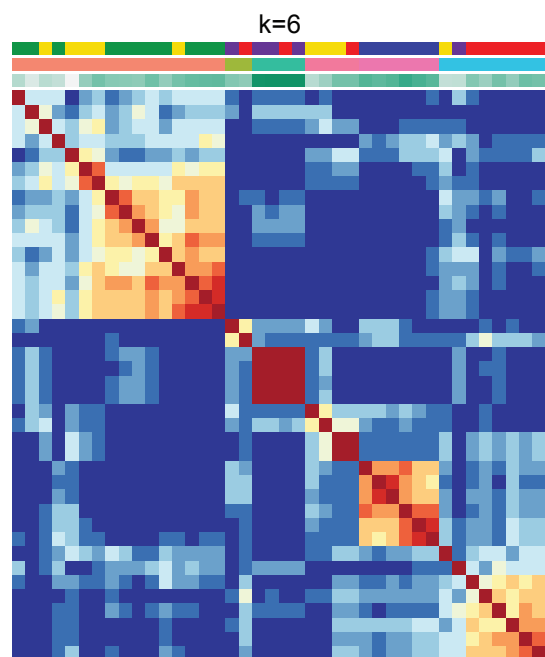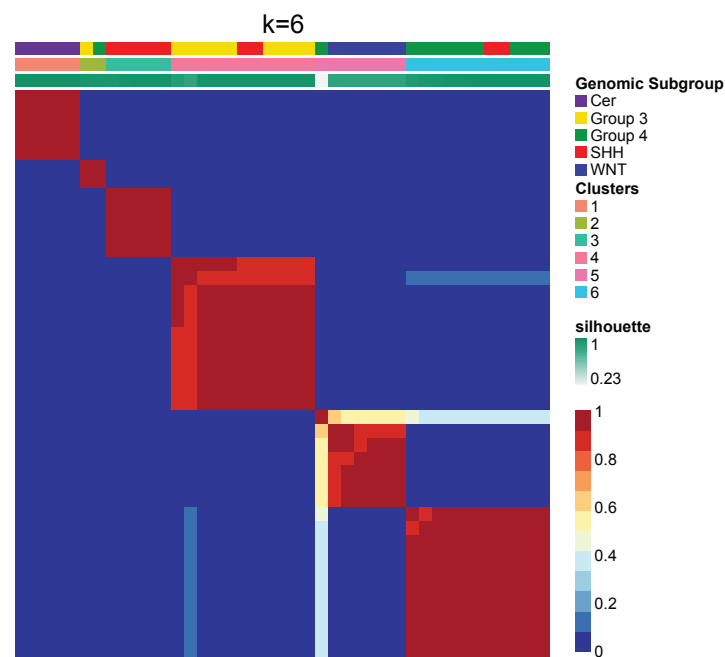

Figure S5

Supplement: Supplementary file 8 — Figure S5. Proteomic subgroup classification recapitulates genomic subgroups using different data elements. Comparison of non-negative matrix factorization consensus clustering between protein and mRNA expression data from 34 primary medulloblastoma and five normal cerebellar tissues. a) The Cophenetic and Silhouette coefficient values for rank k between 2 and 10 in mRNA and protein dataset. b) NMF clusters (k = 6) for the same genes coding for the proteins used in the proteomic classification at the mRNA or protein level. Clustering did not improve for other k values 2 through 10 (data not shown). (PDF 994 kb) [file 40478_2018_548_MOESM8_ESM.pdf]

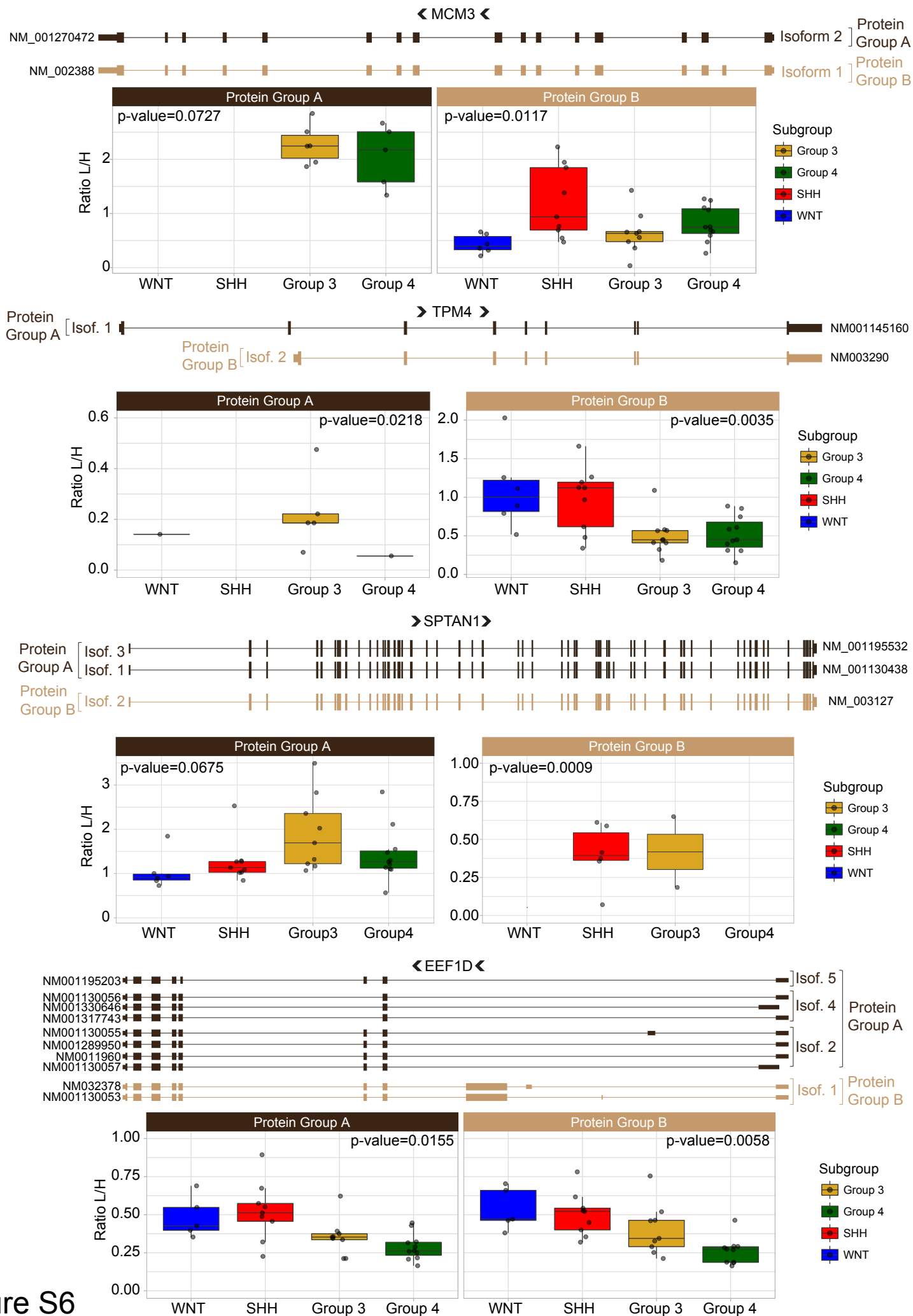

Figure S6

Supplement: Supplementary file 10 — Figure S6. Medulloblastoma subgroup specific isoforms. Schematic representation of MCM3, TPM4, SPTAN1 and EEF1D isoforms. Boxplots show the quantification of each protein isoforms group across all medulloblastoma subgroups. p-values for differences between subgroups were calculated based on the Kruskal-Wallis rank-sum test. A protein group is defined as the group of isoforms that are indistinguishable due to the position of identified peptides. (PDF 1.13 mb) [file 40478_2018_548_MOESM10_ESM.pdf]

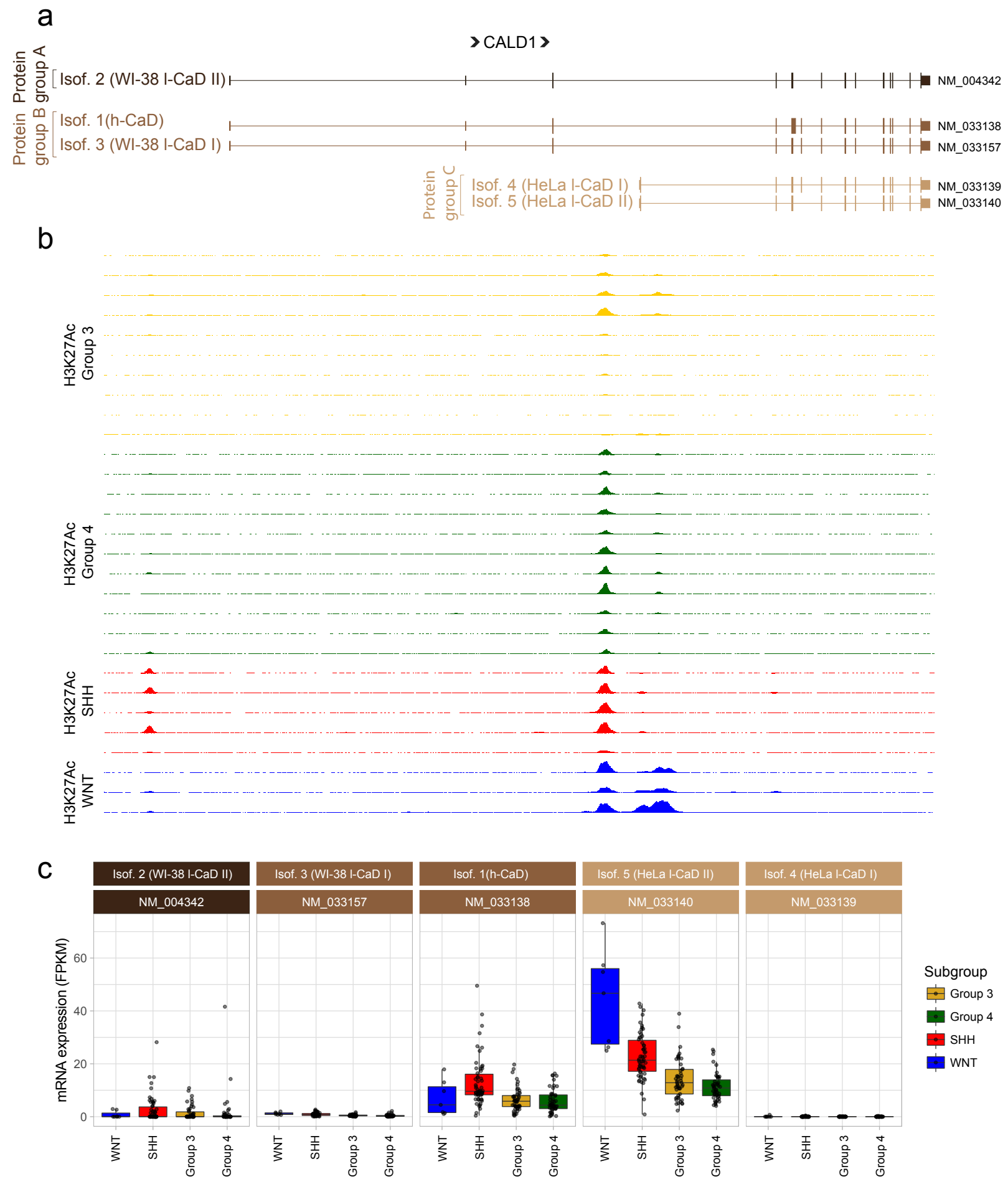

Figure S7

Supplement: Supplementary file 11 — Figure S7. Expression of CALD1 isoforms in medulloblastoma tumors. The protein expression level of CALD1 isoforms in medulloblastoma subgroups is confirmed at the epigenetic (H3K27Ac Chip-seq) and mRNA level. a) Schematic representation of CALD1 isoforms. b) H3K27Ac Chip-seq genome tracks in medulloblastoma tumors. Active transcription region marks (H3K27Ac) are observed in the alternative transcription start site for the isoforms HeLa l-CaD I and II correlating with higher expression of these protein isoforms. c) Boxplots representing the mRNA expression levels for CALD1 isoforms. (PDF 2.2 mb) [file 40478_2018_548_MOESM11_ESM.pdf]

Protein group A

Isoform a

Protein group B

Isoform b

HMGA1

NM\_145899

NM\_145901

NM\_145905

NM\_002131

NM\_145903

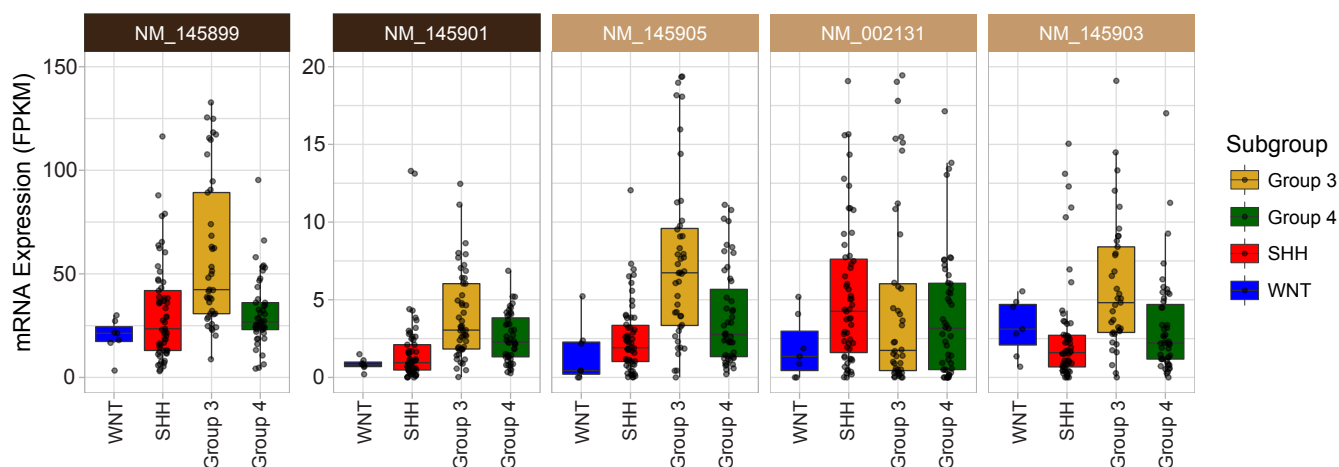

**b**

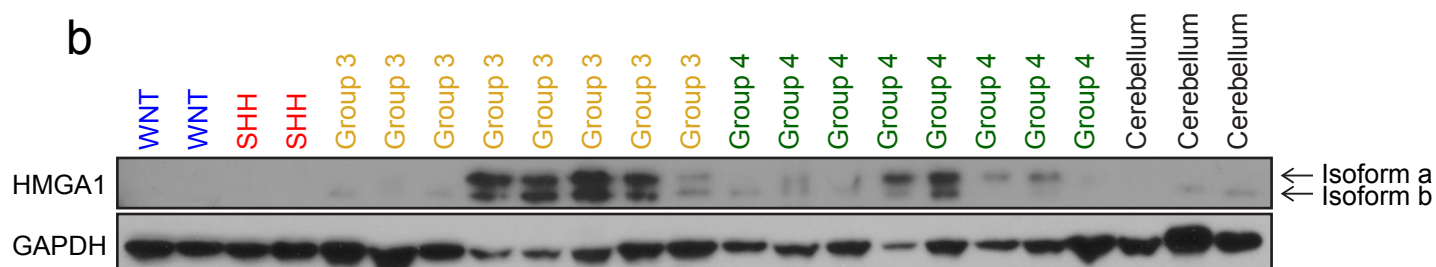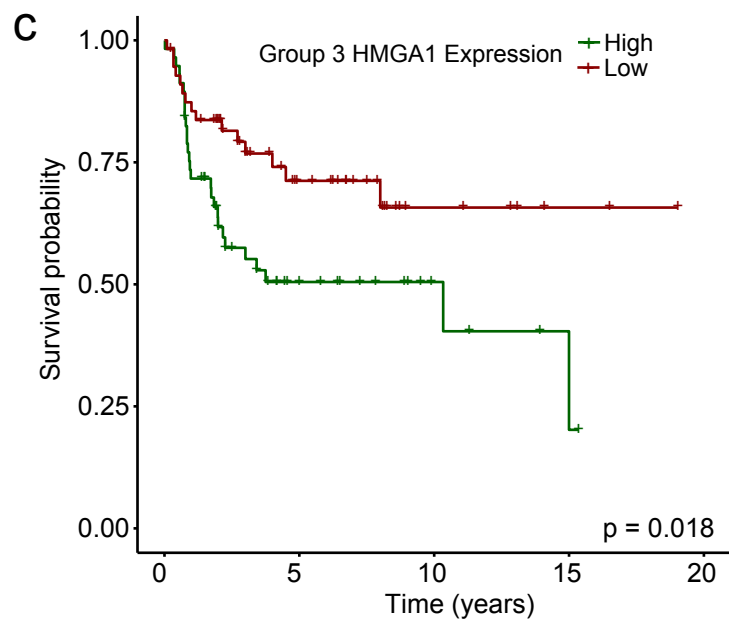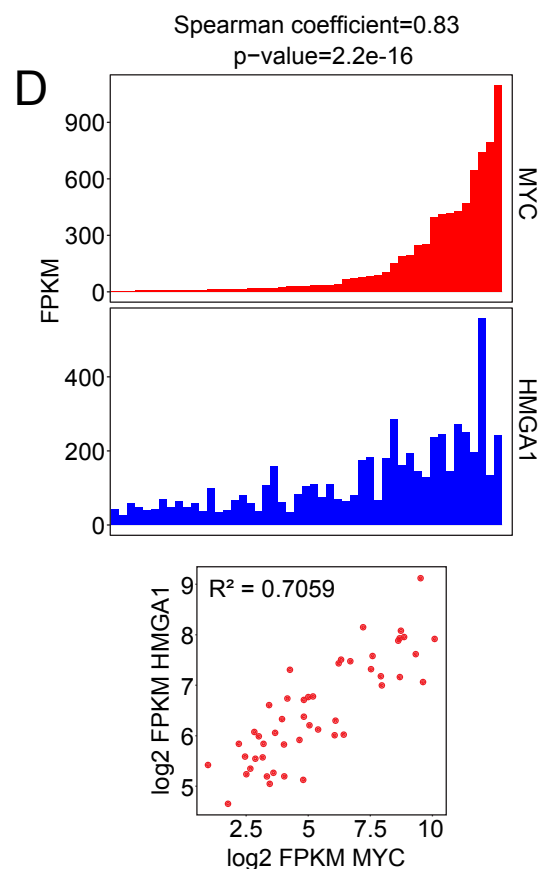

Supplement: Supplementary file 12 — Figure S8. Expression of HMAG1 isoforms in medulloblastoma tumors. a) Schematic representation of HMGA11 isoforms and Boxplots representing the mRNA expression levels for HMGA1 isoforms. b). Western blot of HMGA1 isoforms in the four medulloblastoma subgroups. Both HMAG1 isoforms are highly expressed in group 3 medulloblastoma. c) Kaplan–Meier survival curve shows that increased levels of HMGA1 are associated with poor survival in Group 3 Medulloblastoma. d) Expression level of HMGA1 is highly correlated with the expression of the oncogene MYC in Group 3 Medulloblastoma. (PDF 1.94 mb) [file 40478_2018_548_MOESM12_ESM.pdf]

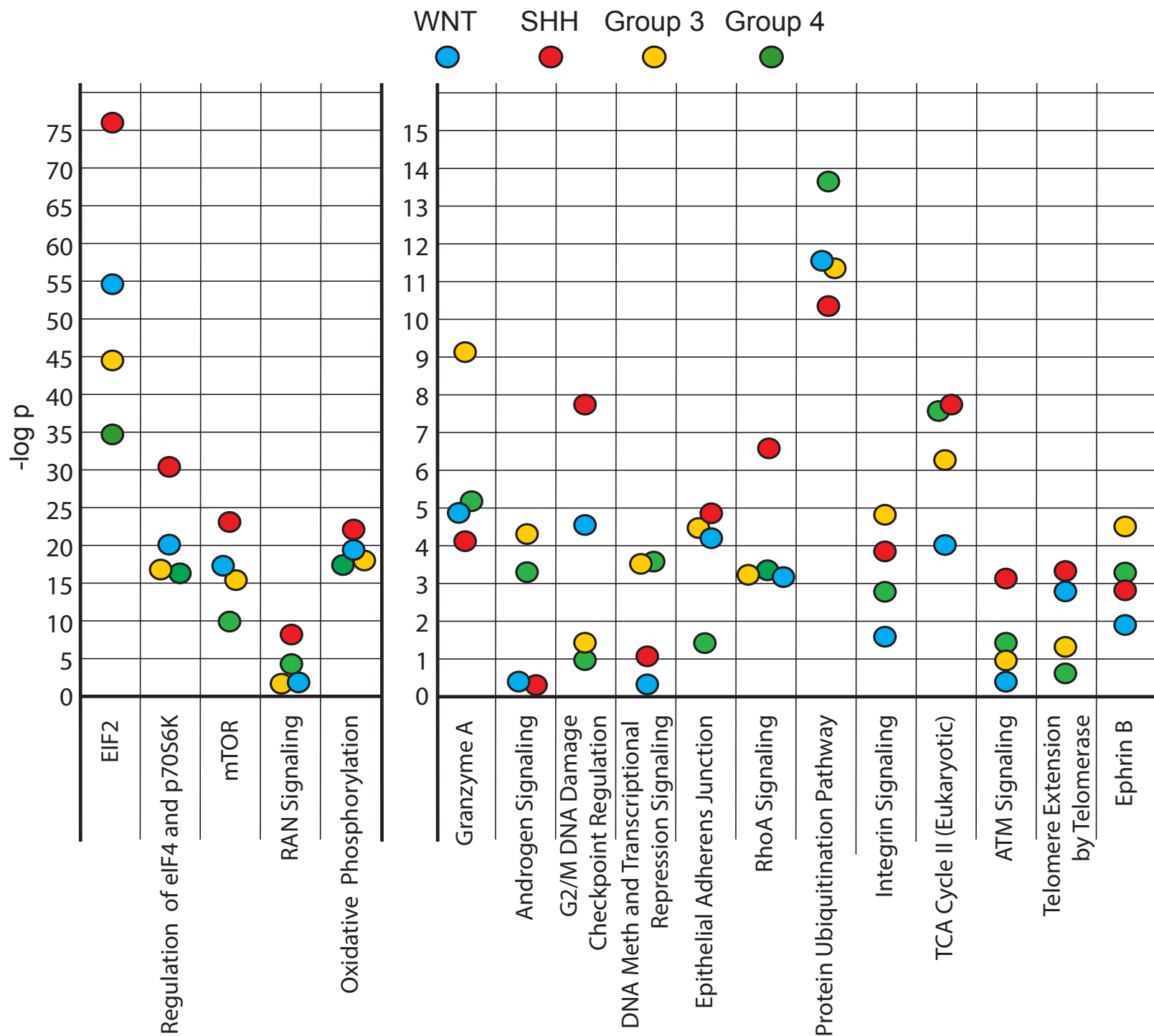

Figure S9

Supplement: Supplementary file 16 — Figure S9. Subgroup representative pathways. Enriched pathways generated with Ingenuity Pathway Analysis software based on lists of differentially quantitated proteins by subgroup normalized to control cerebellum. Each circle plots the p-value for a pathway in each medulloblastoma subgroup. (PDF 623 kb) [file 40478_2018_548_MOESM16_ESM.pdf]

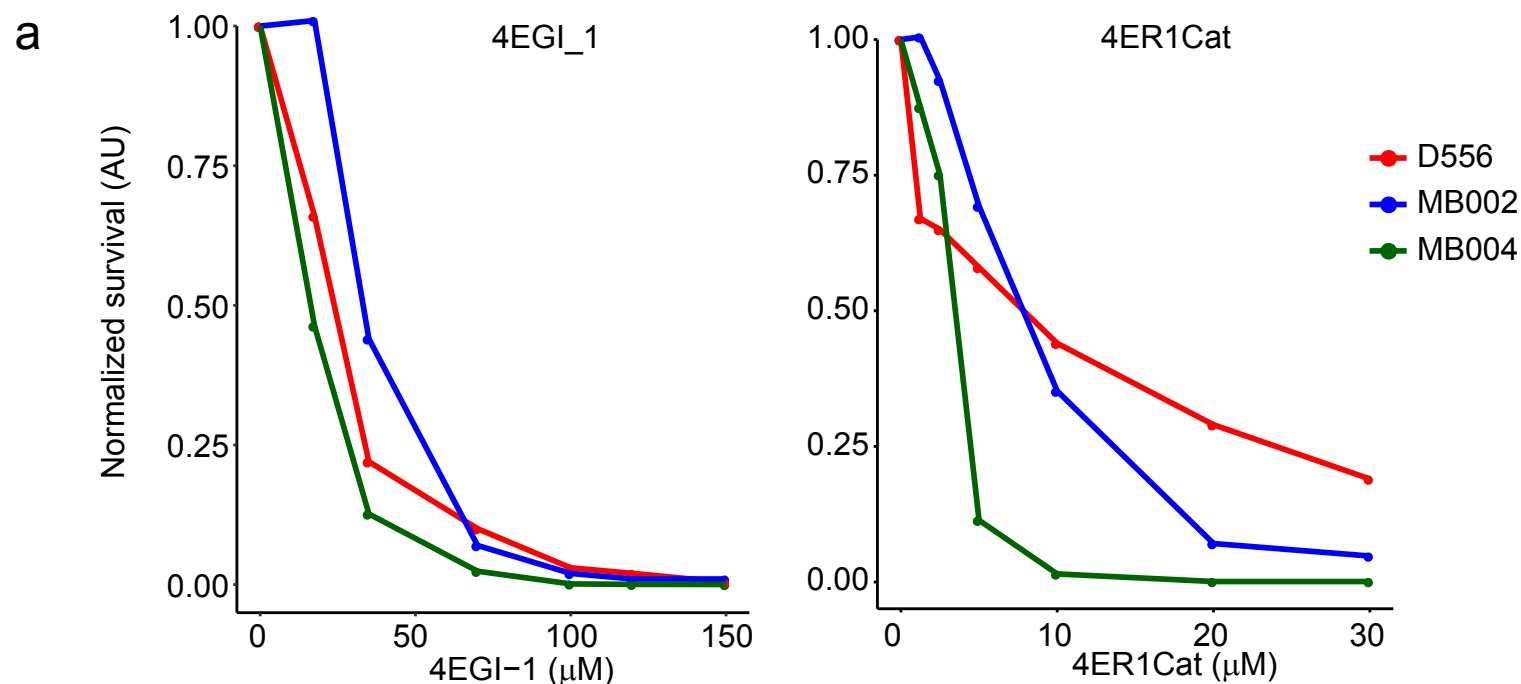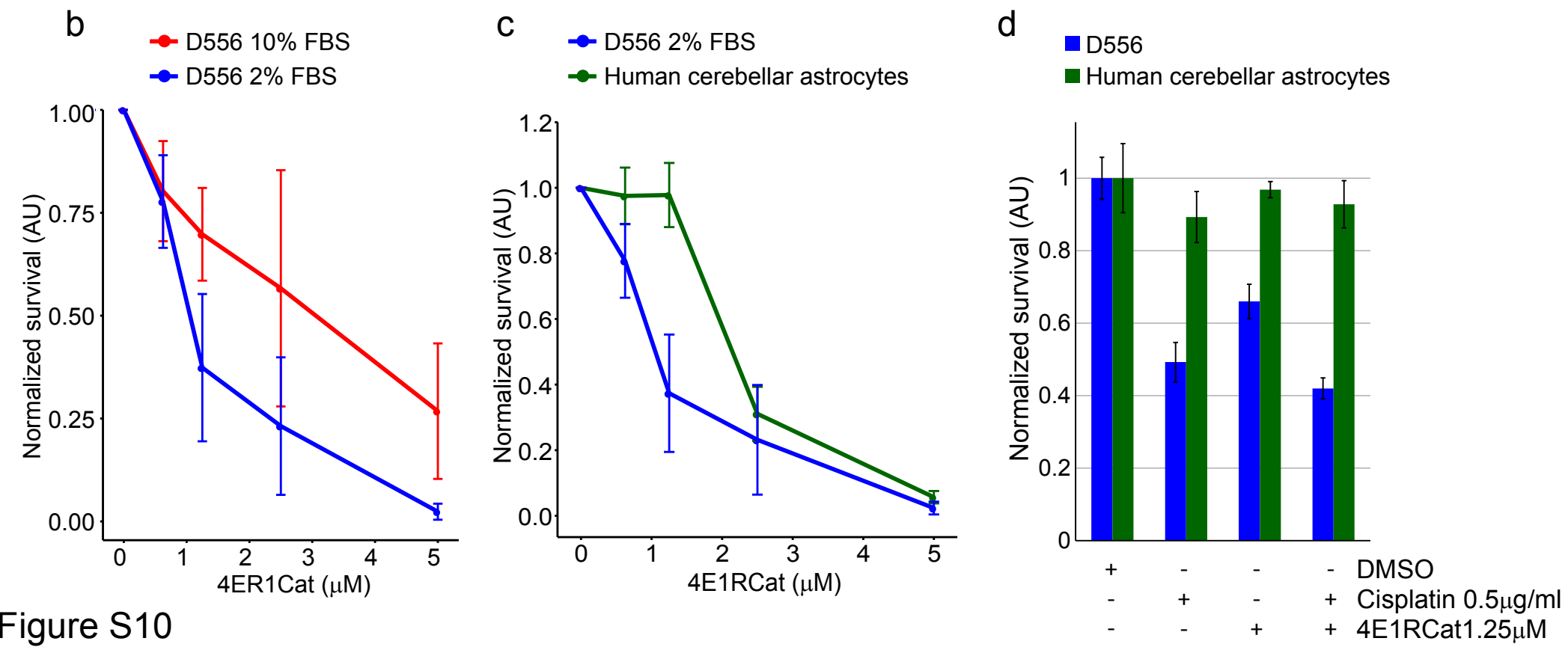

Figure S10

Supplement: Supplementary file 18 — Figure S10. EIF4 inhibitor concentration dependent cell death in medulloblastoma cells. a) MB002, MB004 and D556 cells were treated with the EIF4F inhibitors 4E1RCat for 72 h at the indicated concentrations. In all cell lines we found concentration-dependent cell death when treated with EIF4F inhibitors. D556 at normal (10%FBS) and nutrient deprivation (2% FBS) conditions (b) and D556 and primary human cerebellar astrocytes cells (c) were treated with the EIF4F inhibitor 4E1RCat for 72 h at the indicated concentrations. Error bars indicate the standard variation deviation. d) Treatment of D556 and primary human cerebellar astrocytes cells with 4E1RCat in combination with cisplatin at the indicated concentrations. (PDF 975 kb) [file 40478_2018_548_MOESM18_ESM.pdf]
